# Bioinspired Melanoidin–Polyphenol nanocomplex for hair protection and mechanical reinforcement with antioxidant activity

**DOI:** 10.1016/j.mtbio.2026.103277

**Published:** 2026-05-25

**Authors:** Woo Il Lee, Tae Min Kim, Sanghee Lee, Gibaek Lee, Sung Young Park

**Affiliations:** a Department of IT and Energy Convergence, Korea National University of Transportation, Chungju, 27469, Republic of Korea; b Department of Chemical and Biological Engineering, Korea National University of Transportation, Chungju, 27469, Republic of Korea; c School of Nanomedical Engineering, Korea National University of Transportation, Chungju, 27469, Republic of Korea

**Keywords:** UV-blocking, Melanoidin, Polyphenol, Antioxidant activity, Hair care

## Abstract

Ultraviolet (UV) radiation is one of the most pervasive environmental threats to biological interfaces, driving protein denaturation, structural weakening, and oxidative stress that collectively deteriorate the quality of skin and hair. Despite their widespread use, conventional UV filters are hindered by poor coatability, photoinstability, and safety concerns, underscoring the need for fundamentally new protection strategies. Herein, we report a bioderived melanoidin/tannic acid (MD/TA) nanocomplex that self-assembles into a robust photoprotective coating through synergistic interactions with polyphenols. By leveraging the intrinsic UVA absorption of melanoidin, the broad-spectrum UV shielding of tannic acid, radical-scavenging activity, and interfacial binding, the MD/TA complex forms uniform, durable, and antioxidant-active layers on hair fibers. Beyond surface protection, the coating reprograms oxidative stress responses by suppressing ROS accumulation, thereby restoring cuticle integrity and enhancing tensile resilience. At the molecular level, it activates endogenous antioxidant pathways (*SOD2*, *CAT*) while attenuating apoptosis and inflammatory cascades (*TNF-α*, *IL-1β*) in the presence of UV irradiation. This work establishes a biocompatible, multifunctional photoprotective platform that transcends the limitations of conventional filters by combining durable adhesion with molecular-level antioxidant reprogramming. Thus, the MD/TA nanocomplex exemplifies a synergistic, bio-inspired strategy for long-lasting hair protection against UV-induced oxidative damage.

## Highlights

• Fabrication of photoprotective natural nanocomplex (MD/TA) for hair protection.
• MD/TA combines the UVA and UVB blocking capabilities of melanoidin and tannic acid.
• The MD/TA nanocomplex provides uniform and durable coating on hair strands.
• The nanocomplex provides *in vitro* UV shielding and scavenging of oxidative stress.
• MD/TA provides protection from UV damage by downregulating *TNF-α* and *IL-1β* genes.

## Graphical abstract

**Figure:**
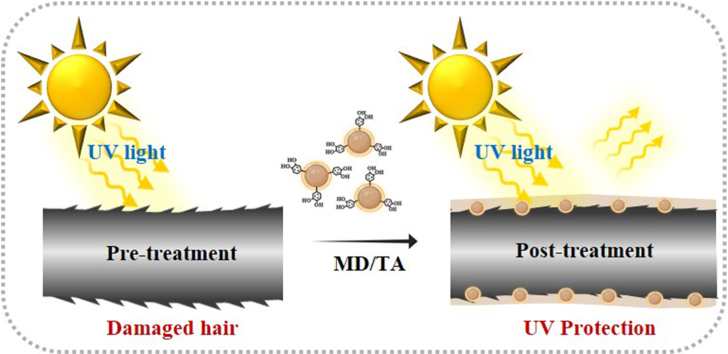

## 1 Introduction

Ultraviolet (UV) exposure significantly contributes to the damage caused not only to the skin but also to the hair, impairing both health and appearance [[1], [2], [3]]. Ultraviolet radiation induces photoaging, pigmentation changes, and loss of elasticity in the skin [[4], [5], [6]]. Hair, lacking a natural protective barrier, is directly exposed to UVA (320–400 nm) and UVB (280–320 nm), which cause various adverse effects [[7], [8], [9], [10], [11]]. For example, UVA penetrates the hair shaft, causing protein degradation and structural weakening; meanwhile, UVB primarily damages the cuticle, leading to roughness, dryness, fading, and reduced elasticity [[12], [13], [14], [15]]. Collectively, such effects reduce the mechanical strength of hair and compromise the color stability of dyed hair. In some cases, they may increase susceptibility to hair loss, ultimately leading to a dull appearance and diminished confidence.

Conventional hair care products have attempted to mitigate UV damage using organic and inorganic filters. Organic compounds (e.g., octinoxate and benzophenone derivatives) absorb UV radiation, whereas inorganic compounds (e.g., ZnO and TiO_2_) reflect and scatter UV light [[16], [17], [18], [19], [20], [21]]. However, these filters are removed during washing, degrade under prolonged sunlight, and pose safety concerns such as skin irritation and endocrine disruption [[22], [23], [24]]. Their inadequate adherence to hair, along with problems such as white casting and a chalky appearance in the case of inorganic compounds, further reduces protective efficacy and adversely impacts cosmetic attributes, including luster, texture, and styling retention.

To address these limitations, natural-based alternatives offering improved safety and multifunctionality have received growing attention. Melanoidin, a polymeric compound formed through the Maillard reaction, exhibits UVA absorption and antioxidant activity [[25], [26], [27]]. However, its sole use is constrained by moderate stability and inadequate UVB absorption, thereby limiting comprehensive photoprotection. Therefore, we developed a complex by incorporating tannic acid (TA), a naturally occurring polyphenol characterized by broad UVA/UVB absorption and potent antioxidant properties [[28], [29], [30]]. During complexation, TA establishes a uniform and stable coating on melanoidin particles, thereby enhancing coating performance and improving UV shielding [31,32]. In addition to providing UV protection, TA interacts with hair proteins *via* hydrogen bonding and affinity interactions, thereby facilitating cuticle repair, enhancing tensile strength, and reducing static charge [[33], [34], [35]]. These restorative functions act synergistically with UV shielding, offering both protective and cosmetic benefits. Therefore, the melanoidin/TA complex constitutes a promising natural photoprotective agent for hair care, offering uniform coating, antioxidant properties, structural stability, and repair capabilities.

In this study, we systematically fabricated and investigated the synthesis of the melanoidin/TA complex, characterized its UVA and UVB absorption spectra, and assessed its coatability and antistatic performance on hair fibers. The synthesis process was facile and comprised of naturally biocompatible compounds like melanoidin and tannic acid. Moreover, we leveraged the cleavage of disulfide bonds in artificial hair samples under UV light leading to the formation of free thiols which further recombined with MD/TA nanocomplex *via* Michael addition reaction leading to the reinforcement of hair and increased mechanical strength compared to commercial hair care products. Furthermore, we evaluated its ability to protect, restore and provide ROS scavenging efficacy in presence of human derived dermal papilla cells (DPCs) which would help to ensure its application as a hair care product in a research and commercial setting.

## 2 Experimental section

### 2.1 Material and characterization

1,3-Dihydroxyacetone (DHA) and L-lysine (Lys) were purchased from TCI (Tokyo, Japan). Tannic acid (TA), phosphate-buffered saline (PBS), 2,2-diphenyl-1-picrylhydrazyl (DPPH), hydrogen peroxide (H_2_O_2_), tris-buffered saline, salicylic acid, ferrous sulfate (FeSO_4_), tris-hydrochloride (tris-HCl), pyrogallol, sodium hydroxide (NaOH), and 3-(4,5-dimethylthiazol-2-yl)-2,5-diphenyltetrazolium bromide (MTT) were obtained from Sigma-Aldrich (St. Louis, MO, USA). Hair samples/wigs (artificial hair, beige; Milbon level 19; Wella chart 15) were purchased from Yeosin (Seoul, Republic of Korea). Murine preadipocyte 3T3-L1 cells were obtained from the Korea Cell Line Bank (Seoul, Republic of Korea). High-glucose Dulbecco's Modified Eagle's Medium (DMEM) purchased from Welgene (Gyeongsan-si, Republic of Korea). Fetal bovine serum (FBS) and penicillin-streptomycin were obtained from Gibco-Invitrogen (Seoul, Republic of Korea). The shampoo base for the coating experiments were fabricated using: sodium C14-16 olefin sulfonate, disodium laureth sulfosuccinate, glycerin, sodium cocoyl isethionate, lauryl hydroxysultaine, butylene glycol, lauryl glucoside, 1-2-hexanediol, *Sesamum indicum* (sesame) seed extract, *Morus nigra* fruit extract, *Tuber melanosporum* extract, *Nigella sativa* seed extract, *Prunus serotina* (wild cherry) fruit extract, *Morus alba* bark extract, niacinamide, Camelia sinensis leaf extract, 1,2,4-trihydroxybenzene, salicylic acid, citric acid, *Mentha piperita* (peppermint) oil, sodium chloride, PPG-3 caprylyl ether, guar hydroxypropyltrimonium chloride, panthenol, polyquartenium-10, ethylhexylglycerin, caprylyl glycol, menthol, caramel).

UV–visible spectra were recorded using an Optizen 2020 spectrophotometer (Mecasys, Daejeon, Republic of Korea). Particle size was measured using a Zetasizer Nano (Malvern Panalytical, Kassel, Germany). SEM images were obtained using a JSM-6700F microscope (JEOL, Tokyo, Japan). Thermogravimetric analysis (TGA) was performed using an SDT 650 analyzer (TA Instruments, New Castle, DE, USA) at a heating rate of 10 °C/min under a nitrogen atmosphere. Fourier-transform infrared (FT-IR) spectra were acquired using a Nicolet iS10 spectrometer (Thermo Fisher Scientific, Waltham, MA, USA). Static contact angles were measured with a KRÜSS DSA100 (KRÜSS GmbH, Hamburg, Germany). Electrical resistivity was measured using a Keithley 2450 source meter (Tektronix Inc., Beaverton, OR, USA). Cyclic voltammetry (CV) was performed using a CorrTest electrochemical workstation (Wuhan, China). Confocal laser scanning microscopy (CLSM) images were acquired using an ECLIPSE Ti2-E microscope (Nikon). Tensile properties were evaluated using a texture analyzer (SurTA 1A; Chemilab Co., Seoul, Republic of Korea). X-ray photoelectron spectroscopy (XPS) data were acquired using an ESCALAB system (PHI Quantera II, Ulvac-PHI, Chigasaki, Japan).

### 2.2 Synthesis of UV-blocking materials melanoidin/tannic acid (MD/TA)

Melanoidin was synthesized by reacting 9 g of DHA and 5.5 g of Lys in 20 mL of double-distilled water (DDW) at 50 °C for 8 h, followed by freeze-drying to yield a brown solid [36]. Subsequently, 0.725 g of the dried melanoidin was dissolved in 1 mL of water, and 2 mL of 1 M NaOH was added to adjust the pH to 9–10. Separately, 75 mg of TA was dissolved in 1 mL of DDW and added to the melanoidin solution. The mixture was stirred for 12 h, then frozen and freeze-dried to obtain the final MD/TA product. For the demonstration of the colloidal stability of the MD and MD/TA nanocomplex, they were exposed to PBS pH 6.0, 7.0, and 8.0. Additionally, the ionic colloidal stability was analyzed in the presence of 50 mM NaCl and KCl and PBS solutions respectively [37]. The concentration employed was 10 mg/mL and corresponding UV spectra measurements at 0 and 7 days were carried out at 0.05 mg/mL.

### 2.3 Investigation of electrical properties and coating potential of MD/TA nanocomplex

To evaluate the electrical performance, MD or MD/TA was dissolved in Tris-buffered saline (TBS, pH 8.5) at a concentration of 10 mg/mL. Silicon wafers (1 cm × 1 cm) were immersed in the solution for 12 h and then dried at room temperature. The coated wafers were used as working electrodes for cyclic voltammetry (CV), with Ag/AgCl electrode as the reference electrode and a platinum wire as the counter electrode. CV was conducted at a scan rate of 50 mV/s over a potential range of −2.5 to +2.5 V (vs. Ag/AgCl). Electrical resistance was measured using a Keithley 2450 source meter under a bias voltage of 1 V in a two-electrode configuration [38]. The static contact angle of the coated surface was analyzed using a KRÜSS DSA100 goniometer, applying the Cecil Drops method. All measurements were performed in triplicate (n = 3). The coating potential of MD and MD/TA nanocomplex was further investigated by mixing them (2.31 g) into shampoo base (30 g). This mixture was then mixed with 10 mg Rhodamine B dye. Following this, the MD and MD/TA mixed Rhodamine B labelled shampoo mixture was applied on artificial hair strands of thickness: 0.04 ± 0.01 mm for 3 min. This was followed by washing the sample for 2 min and rinsed. The dried samples were then observed under CLSM to analyze the coating efficiency. Furthermore, the dried hair samples were subjected to UV treatment and exposed to UV radiation (∼365 nm) for 1 day and analyzed for elemental mapping (EDX).

### 2.4 Investigation of UV blocking properties of MD/TA nanocomplex

For the Ellman's assay analysis to determine UV blocking effect: artificial hair samples of thickness: 0.04 ± 0.01 mm was applied with MD and MD/TA nanocomplex sample mixed in shampoo base for 3 min. This was followed by washing the sample for 2 min and rinsed. The samples were dried for 2-3 min and exposed to UV radiation (∼365 nm) for 1 day. Then DTNB (4 mg) was dissolved in 1 mL of sodium dodecyl sulfate (SDS) aqueous solution (2%) and 90 μL of the resulting solution was diluted in 4.91 mL of SDS solution (2%) to prepare a DTNB solution. We immersed the control, MD and MD/TA coated hair samples before and after treatment with UV light in the DTNB solution and incubated it for 15 min at room temperature after covering with an Al foil. The TNB concentration (formed by the interaction between DTNB and residual thiol from the hair strands were then measured and calculated by comparing the absorbance peaks at 412 nm based on the linear calibration curve [39].

### 2.5 Application of the MD/TA solution to hair samples and evaluation of mechanical properties

The MD and MD/TA samples were dissolved in shampoo base with gentle stirring. The solution was then uniformly applied onto artificial hair samples (thickness: 0.04 ± 0.01 mm) and for 3 min. This was followed by washing, rinsing and drying the sample for 3 min. The coated samples were then divided into before UV treatment and after UV treatment. The after UV treatment samples were irradiated with UV light (∼365 nm) for 1 day. The before and after UV treated hair strands were then mounted between two grips spaced 13.5 cm apart and subjected to uniaxial tensile testing at a strain rate of 10 mm/s using a texture analyzer. The tensile test was repeated three times per sample (n = 3) to evaluate the mechanical performance. Additionally, for the rinse test to analyze the durability of the coating and repair properties of MD and MD/TA, the artificial hair samples of thickness: 0.04 ± 0.01 mm were applied with MD and MD/TA nanocomplex sample mixed in shampoo base for 3 min. This was followed by washing the sample for 2 min and rinsed. The samples were dried for 2-3 min and this whole process was repeated for 10 cycles. These samples were then subjected to UTM based mechanical property analysis and SEM optical imaging at various rinse test cycles.

### 2.6 Determination of reactive oxygen species (ROS)-scavenging performance of MD/TA

The reactive oxygen species (ROS) scavenging activities of MD and MD/TA were evaluated *via* DPPH, hydroxyl radical (•OH), and superoxide anion (O_2_^•-^) assays [40]. For the DPPH assay, 3 mL of 0.1 mM DPPH solution was mixed with 3 mL of the nanocomplex solution and incubated at 37 °C for 30 min. The absorbance was measured at 517 nm using a UV–vis spectrophotometer. For •OH scavenging, 0.5 mL of the nanocomplex solution was added to a mixture of salicylic acid (6 mM, 1 mL), PBS (pH 7.4, 1.5 mL), FeSO_4_ (6 mM, 1 mL), and H_2_O_2_ (0.01%, 0.5 mL). After incubation at 37 °C for 30 min, the absorbance was measured at 510 nm. For O_2_^•-^ scavenging assay, 0.5 mL of the nanocomplex solution was mixed with 3 mL of Tris-HCl buffer (pH 8.2) and 0.8 mL of 0.05 M pyrogallol. The reaction was carried out at 25 °C for 5 min, followed by the addition of 1 mL of 8 M HCl. The absorbance was measured at 325 nm. The ROS scavenging efficiency (%) was calculated using the following equation:

$$\text{ROS}\;\text{scavenging}\;\text{performance}\;\left(\%\right)=\left(\left(A_{\text{std}}\;‐\;A_{\text{sam}}\right)/A_{\text{std}}\right)\times 100\%$$

where A_std_ is the absorbance of the control (blank) and A_sam_ is the absorbance of the sample.

### 2.7 Cell culture, RNA isolation, and quantitative real-time PCR (qRT-PCR)

Murine preadipocyte 3T3-L1 cells were obtained from the Korea Cell Line Bank (Seoul, Republic of Korea) and cultured in high-glucose Dulbecco's Modified Eagle Medium (DMEM) supplemented with 10% fetal bovine serum (FBS), 100 U/mL penicillin, and 100 μg/mL streptomycin at 37 °C in a humidified atmosphere containing 5% CO_2_. Total RNA was extracted using RNAiso Plus reagent (#9109, Takara Bio, Japan), and complementary DNA (cDNA) was synthesized using the 5× All-In-One RT Master Mix (PrimeScript™ RT Master Mix, RR036A, Takara). Quantitative real-time PCR (qRT-PCR) was performed using TB Green® Premix Ex Taq™ II (RR830A, Takara) on a QuantStudio 3 Real-Time PCR System (Thermo Fisher Scientific, USA). All reactions were carried out in triplicate. Gene expression was normalized to *β-actin*, and the primer sequences used were as follows: *β-actin*: Forward: 5′-GGC ATC CTC ACC CTG AAG TA-3′; Reverse: 3′-AGG TGT GGt GCC AGA TTT TC-5′. *SOD2*: Forward: 5′-CTG GAC AAA CCT CAG CCC TAA C-3′; Reverse: 3′-CAA CCA GGC TCA GGT TGT TTA A-5′. *Catalase (CAT)*: Forward: 5′-GTG CGG AGA TTC AAC ACT GCC A-3′; Reverse: 3′-CGG CAA TGT TCT CAC ACA GAC G-5′. *TNF-α*: Forward: 5′-CTC TTC TGC CTG CTG CAC TTT G-3′; Reverse: 3′-GAG TGA CAA GCC TGT AGC CCA T-5′. *IL-1β*: Forward: 5′-TCC AGG ATG AGG ACA TGA GCA C-3′; Reverse: 3′-GAA CGT CAC ACA CCA GCA GGT TA-5′.

### 2.8 In vitro analysis of UV-blocking properties

To verify the UV-blocking effect *in vitro*, MD or MD/TA was first dissolved in TBS (pH 8.5) at a concentration of 10 mg/mL. Subsequently, the PET films were immersed in the solution and coated for 12 h, followed by thorough washing and drying. The coated films were placed on a plate seeded with cells, and irradiated with UV light (wavelength: 365 nm, duration of UV treatment: 0, 0.5, 1, 4 and 12 h) for a specified period. Subsequently, the viability of the cells was assessed using CLSM live and dead assay staining and evaluated using the MTT assay and RT-PCR.

To evaluate the cytotoxicity, 3T3-L1 cells at a density of 5 × 10^5^ cells/well were cultured for 24 h in a 37 °C incubator in a humidified atmosphere containing 5% CO_2_. Stock solutions of MD or MD/TA were prepared in DMEM medium. The culture medium was replaced with the stock solution and incubated overnight. After removal of the stock solution, MTT solution was added. Cell viability was evaluated by measuring the absorbance at 570 nm using a microplate reader.

Further, flow cytometry was performed by culturing 3T3-L1 cells at a density of 5 × 10^5^ cells/well in 6-well plates for 24 h at 37 °C (5% CO_2_). The medium was then replaced with the sample stock solution, followed by incubation for the designated time. The samples were collected and dissolved in Annexin V Binding Buffer (10×) (pH 7.4) and PI, followed by evaluation at an excitation wavelength of 488 nm and emission wavelengths of 530/30 nm and 574/26 nm, respectively.

The *in vitro* cytotoxicity of MD and MD/TA was evaluated using 3T3-L1 cells. After preparing a cell suspension at a concentration of 5 × 10^5^ cells/mL, 50 μL of the sample stock solution was applied to a CLSM dish and mixed with 100 μL of the cell suspension. The mixture was cultured for 12 h at 37 °C in a humidity-controlled 5% CO_2_ environment. After incubation, the hydrogel was washed with 100 μL of phosphate-buffered saline (PBS), stained with 100 μL of Calcein AM for 30 min, and then incubated with 50 μL of propidium iodide (PI) for 10 min. The stained hydrogel was observed by CLSM at 100 μm magnification to identify live and dead cells.

### 2.9 In vitro analysis of biocompatibility, UV blocking and ROS scavenging potential of MD/TA nanocomplex in presence of dermal papilla cells (DPCs)

The dermal papilla cells (DPCs) were cultured in high-glucose Dulbecco's Modified Eagle Medium (DMEM) supplemented with 10% fetal bovine serum (FBS), 100 U/mL penicillin, and 100 μg/mL streptomycin at 37 °C in a humidified atmosphere containing 5% CO_2_. Once, the cells reached 80-90% confluency, they were detached using trypsin-EDTA and the pellet obtained was used to make cell suspension of cell density 5 x 10^5^ cells/mL. Then various experiments like cytotoxicity assay using various concentration for MD/TA and UV blocking capability (similar protocol as for 3T3-L1 cells) using 1 mg/mL of the MD/TA sample was analyzed using a microplate reader (MTT). Additionally, the live and dead analysis using CLSM was analyzed using 5 × 10^5^ cells/mL, 100 μL of the sample stock solution was applied to a CLSM dish and mixed with 200 μL of the cell suspension. The mixture was cultured for 12 h at 37 °C in a humidity-controlled 5% CO_2_ environment. After incubation, the hydrogel was washed with 100 μL of phosphate-buffered saline (PBS), stained with 100 μL of calcein AM for 30 min, and then incubated with 50 μL of propidium iodide (PI) for 10 min. The stained hydrogel was observed by CLSM at 100 μm magnification to identify live and dead cells. A similar procedure was followed for the UV blocking: live and dead analysis using CLSM. To check the ROS scavenging capability of MD/TA in presence of DPCs, the cells were induced with 300 μM H_2_O_2_ for 3 h followed by a similar treatment pattern with MD and MD/TA samples. The cells were then stained with 2′,7′-dichlorodihydrofluorescein diacetate (H_2_-DCFH-DA) dye for a period of 30 min and observed under a CLSM.

### 2.10 Statistical analysis

All data are presented as the mean ± SD of the mean from a minimum of n = 3 experiments. Statistical analysis was calculated in Microsoft excel, Microsoft office 2021 using unpaired Student's t-test, and significance was defined as ∗p < 0.05; ∗∗p < 0.01; ∗∗∗p < 0.001; ∗∗∗∗p < 0.0001; ns indicates non-significant differences.

## 3 Results and discussion

### 3.1 Design and characterization of MD/TA nanocomplex for UV protection and hair reinforcement

**Fig. 1:**
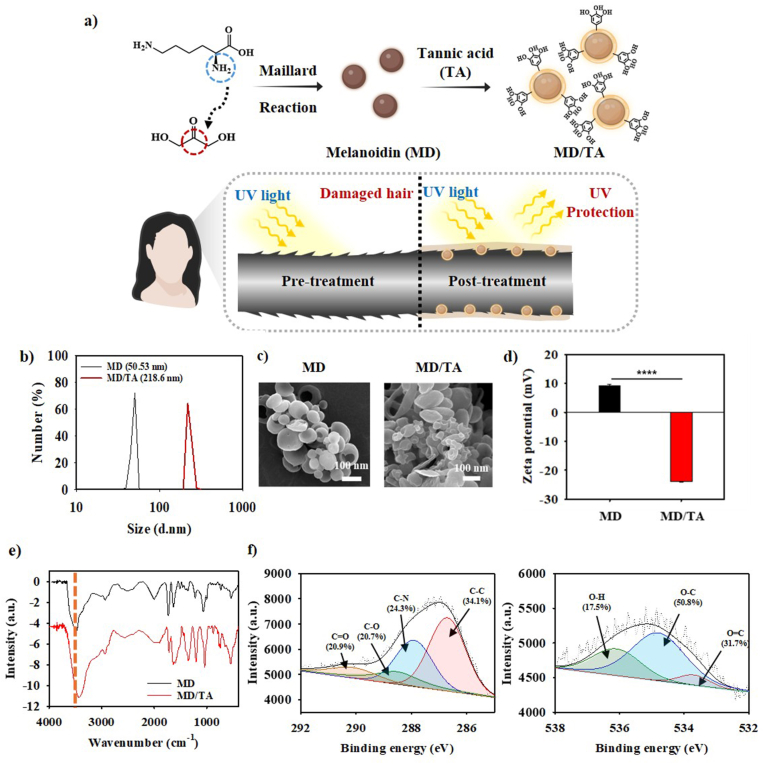
**Characterization and UV-Protective mechanism of MD/TA nanocomplex.** (**a**) Schematic illustration of the synthesis of melanoidin/tannic acid (MD/TA) nanocomplex *via* Maillard reaction and the protective effect of MD/TA on hair under UV exposure. (**b**) Particle size distribution, (**c**) SEM images, (**d**) zeta potential surface charge analysis for successful complexation, (**e**) FT-IR spectra of MD and MD/TA. (**f**) X-ray photoelectron spectroscopic (XPS) analysis of C*1s* and O*1s* spectra following formation of MD/TA nanocomplex. All analysis was carried out for n = 3 samples. An unpaired Student's t-test was used for statistical analysis. ∗p < 0.05; ∗∗p < 0.01; ∗∗∗p < 0.001; ∗∗∗∗p < 0.0001; ns indicates non-significant differences.

A functional material for UV protection and hair care was developed using a melanoidin/tannic acid (MD/TA) complex. In this formulation, dihydroxyacetone (DHA) reacts with lysine (Lys) *via* the Maillard reaction to form melanoidin (MD), which is complexed with tannic acid (TA), a naturally occurring polyphenol with UV absorption and antioxidant activity. The MD/TA system not only absorbs UV radiation but also firmly adheres to the hair surface *via* polyphenol chemistry (numerous galloyl and catechol functionalities on the MD/TA surface), forming a uniform and durable protective coating. This coating functions as a physical barrier that mitigates external stress, minimizes cuticle disruption, and improves overall cosmetic properties. As schematically illustrated in Fig. 1a, untreated UV-exposed hair exhibited pronounced cuticle roughness and damage, whereas hair treated with MD/TA exhibits a smoother and reinforced structure. These results demonstrate that the MD/TA complex contributes to both photoprotection and the preservation of hair integrity and appearance.

In order to comprehend the characteristics of the nanocomplex, a preliminary comparison of the particle sizes was conducted (Fig. 1b). Only melanoidin exhibited a particle size of 50.53 nm, which increased to 218.6 nm upon combination with TA. Furthermore, SEM analysis demonstrated that the MD/TA particles exhibited more pronounced aggregation compared to the MD particles (Fig. 1c). This phenomenon was attributed to the formation of hydrogen bonds between MD and TA, thereby leading to the formation of the MD/TA nanocomplex. Additionally, the UV-vis spectroscopic analysis of the MD and MD/TA nanocomplex was analyzed which showed the presence of peak at ∼275 nm for melanoidin (Fig. S1), whereas the MD/TA nanocomplex showed similarities with the absorbance spectra of TA and an increase in the absorbance intensity of peak at ∼275 nm due to the presence of galloyl groups. Subsequently, the surface charge of the particles was examined in Fig. 1d. It was confirmed that MD particles (9.1 mV) carried a positive charge due to the amine group of Lys, while MD/TA particles (−24.0 mV) carried a negative charge due to the -OH in the galloyl group of TA. Furthermore, the presence of hydrogen bonds in MD and MD/TA was confirmed by FT-IR spectroscopy, which showed a shift in the O-H bond stretching frequency from 3482 cm^−1^ to 3422 cm^−1^ (Fig. 1e) which suggests the occurrence of hydrogen bonding [41]. From the results obtained *via* SEM, zeta potential, and FT-IR, it can be assumed that the presence of electrostatic interactions and hydrogen bonding cooperatively influenced the assembly of MD and TA into a stable nanostructure thereby leading to the formation of MD/TA nanocomplex. XRD analysis demonstrated that both MD and MD/TA possessed a broad peak around 2θ = 20°, indicating their amorphous structure (Fig. S2). Furthermore, the thermal stability of the samples was assessed by means of TGA measurements. The results obtained from these measurements revealed that MD/TA nanocomplex exhibited significantly higher thermal stability in comparison with MD, thereby indicating that the comparatively poor stability of MD is stabilized by electrostatic interactions and H-bonding after introduction of TA (Fig. S3). The successful synthesis of MD and MD/TA complexes was confirmed through XPS analysis as illustrated in Fig. 1e. In the XPS survey spectra in Fig. S4a and Fig. S4b, the C*1s*, N*1s*, and O*1s* peaks are identified, wherein, the O*1s* peak showed a significant increase in the case of MD/TA, indicating an increase in oxygen content as a result of the introduction of TA. Furthermore, the MD C*1s* spectra in Fig. S4c, demonstrates the presence of C–C, C–N, C=O, and C–O bonds in their respective ratios, thereby indicating that the organic skeleton of the MD structure is well formed. The O*1s* spectrum deconvolution of MD reveals the presence of various oxygen-based functional groups, as evidenced by the O–C, O–H, and O=C peaks. Finally, the colloidal stability of the MD/TA nanocomplex was analyzed for a period of 7 days in various pH solutions. It can be seen from Fig. S5, that the MD/TA remains in solution devoid of an aggregation demonstrating absence of any interaction. Moreover, the combined pH and ionic colloidal (NaCl, KCl, and PBS) stability of the MD/TA nanocomplex showed almost no variance in its absorbance intensity at 275 nm when checked for UV analysis as shown in Fig. S6. This in combination with the previous results obtained from various analyses provide the confirmation of the successful formation of MD/TA nanocomplex after the reaction of DHA and Lys giving MD followed by the complexation between MD and TA.

**Fig. 2:**
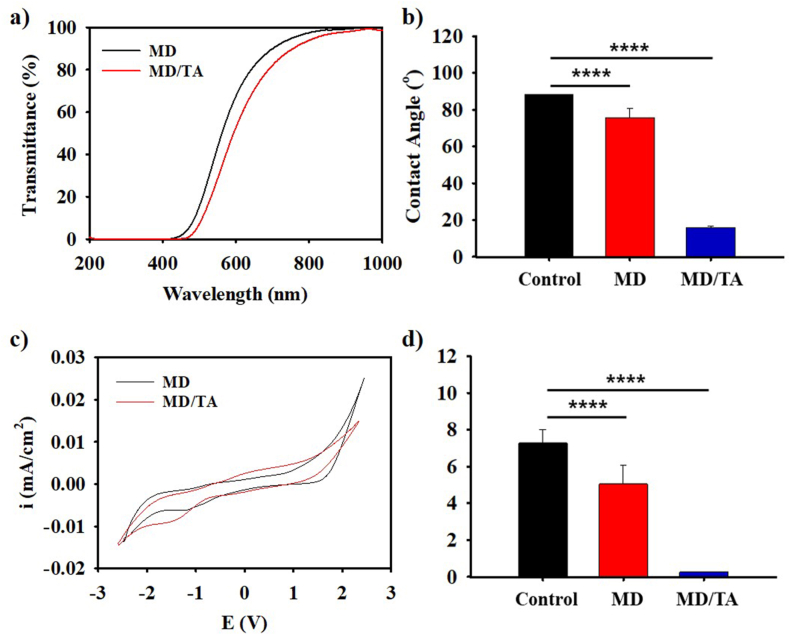
**Photoprotective and electrochemical properties of MD/TA.** (**a**) Comparison of UV-vis spectrum of MD and MD/TA solution. (**b**) Contact angle, (**c**) cyclic voltammetry (CV), and (**d**) sourcemeter resistance analysis of control (bare Si-wafer), MD, and MD/TA coated Si-wafer. All analysis was carried out for n = 3 samples. An unpaired Student's t-test was used for statistical analysis. ∗p < 0.05; ∗∗p < 0.01; ∗∗∗p < 0.001; ∗∗∗∗p < 0.0001; ns indicates non-significant differences.

To evaluate the UV-shielding performance, the optical transmittance of each sample was analyzed using UV–visible spectroscopy (Fig. 2a). The investigation revealed that MD/TA exhibited superior UV protection in comparison to MD. Although melanoidin alone exhibits moderate UV-blocking ability due to its π-conjugated structure, the addition of tannic acid (TA), a polyphenolic compound with strong UV-absorbing capability, greatly increases the absorption range and enhances overall UV protection. This synergistic effect results from the combined photophysical properties of both components, particularly in the UVA and UVB regions. Subsequent UV protection testing was conducted on the MD and MD/TA samples, as well as the control (DDW). This involved the transmission of light through each solution and the evaluation of the UV transmittance on a paper screen. As demonstrated in Fig. S7, the transmission of UV light through a paper screen after passing through a glass cuvette containing DDW can be observed. For MD and MD/TA, the UV protection properties demonstrated that no such UV light penetrated the paper screen behind the solution. These findings showed that the MD/TA complex can act as a robust photoprotective coating in the presence of UV light exposure, thereby holding promise for UV-blocking based cosmetic applications.

Following the assessment of its UV-protection efficiency, the efficiency of MD and MD/TA surface coating was analyzed. MD and MD/TA were coated on Si wafers, and contact angle measurements were performed (Fig. 2b). The contact angle value of 15.9° was found to be significantly different for the Si-wafer coated with MD/TA compared to the control (88.2°) and MD (75.7°). The corresponding images for the contact angle measurements as given in Fig. S8 corroborate the above findings, this indicates that the MD/TA nanocomplex was coated uniformly due to the effect of TA. Additionally, the coating capability of the MD and MD/TA samples on artificial hair was analyzed by observing the FL signals from confocal laser scanning microscopy (CLSM) images after mixing with Rhodamine B dye. From the images in Fig. S9, it can be seen that the control sample didn't show any FL signals, whereas the MD coated sample showed slight red FL signals as compared to the bright red LF signals of MD/TA. This was caused by the influence of phenolic rich galloyl groups of tannic acid in the MD/TA which forms multiple hydrogen bonds and hydrophobic interactions with the various hair proteins thereby helping in the coating process [[42], [43], [44]]. Moreover, the elemental analysis of the untreated hair strands, MD and MD/TA coated hair strands after irradiation with UV light (∼365 nm) for 1 day were performed using EDX spectral analysis. The results in Fig. S10 showed the presence of C, O, N and S in the hair strands. Furthermore, a higher amount of Sulphur in the control owing to the abundant -SH groups caused by the cleavage of -S-S- bonds in keratin is observed in comparison to MD and MD/TA coated samples. This may have been influenced by the presence of galloyl moieties in the MD/TA which undergoes Michael addition reaction with the -SH groups demonstrating efficient coating and damage repairing capability [45,46]. Moreover, the coating durability of the MD and MD/TA nanocomplex on artificial hair strands was analyzed by washing and rinsing for 10 cycles. From the SEM images in Fig. S11, it can be seen that the control showed disruption of the surface morphology of the cuticles as compared to MD and MD/TA coated hair strand, with the MD/TA coated sample appearing to possess a smoother surface even after 10 washing and rinsing cycles. Similarly, the UTM analysis as shown in Fig. S12, showed that the MD/TA coated hair strands showed similar strain % even after 10 washings. This further verified our analysis about the hair coating ability of the MD/TA nanocomplex and its protective capabilities. Additionally, Fig. 2c shows the cyclic voltammetry (CV) curves of MD and MD/TA coated Si-wafer measured between −2.5 V and +2.5 V. Both samples exhibit redox behavior, indicating the presence of electroactive functional groups. Compared to MD, the MD/TA complex shows a slightly broader current response and a more symmetric curve, suggesting enhanced charge-transfer capability and stable redox reversibility introduced by the polyphenolic structure of tannic acid. The electrical resistance of MD and MD/TA-coated Si-wafer was measured, and the results showed that MD/TA has superior electrical conductivity when compared to MD (Fig. 2d). This occurrence can be attributed to the polyphenolic structure of TA, which has been demonstrated to facilitate electron transfer. From a functional perspective, the improvement in conductivity in case of MD/TA coating highlights its scope for the availability of electrons which can also mitigate oxidative stress *via* its catalytic activity on the hair surface by efficiently donating electrons to reactive oxygen species (ROS), further reinforcing its dual role as both a physical and chemical protector.

### 3.2 Performance of MD/TA in hair UV shielding and restoration

**Fig. 3:**
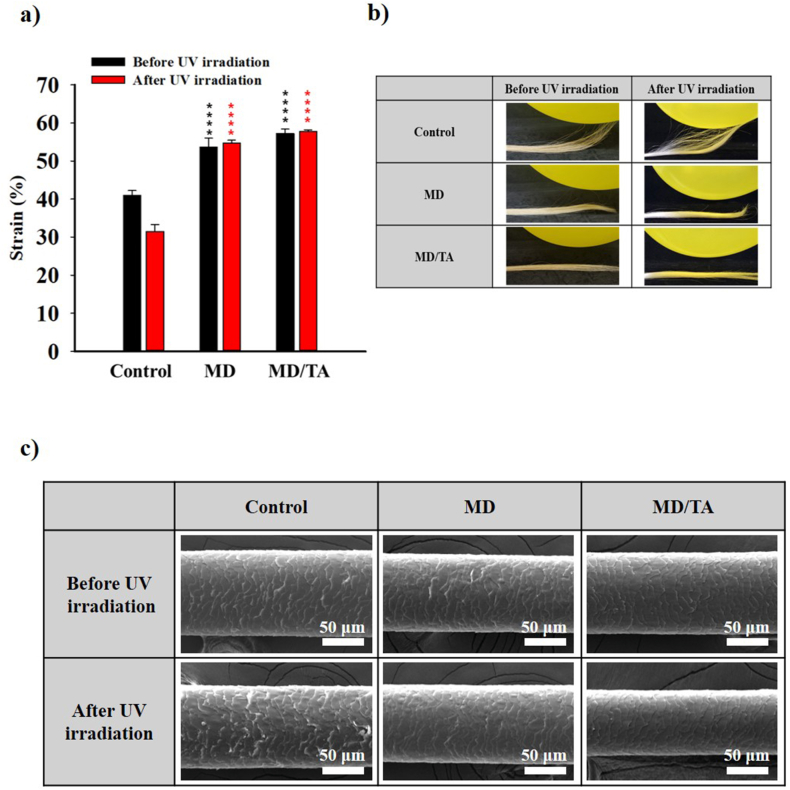
**Mechanical and surface protective effects of hair treated with MD/TA under UV exposure.** (**a**) Tensile strength analysis, (**b**) antistatic test, and (**c**) SEM images of MD and MD/TA nanocomplex treated hair samples before and after UV irradiation. All samples analyzed for n = 3 samples. An unpaired Student's t-test was used for statistical analysis. ∗p < 0.05; ∗∗p < 0.01; ∗∗∗p < 0.001; ∗∗∗∗p < 0.0001; ns indicates non-significant differences.

The effect of MD/TA on the mechanical strength of hair fibers was evaluated through tensile testing. As shown in Fig. 3a, the control group exhibited a relatively low tensile strength (41.0%) before UV exposure. In contrast, MD- and MD/TA-treated samples showed significant improvements, reaching approximately 53.7% and 57.2%, respectively before UV exposure. The enhanced performance in the MD/TA group is likely due to the effective surface coating, as TA promotes stronger adhesion and uniform coating formation on the hair fiber, thereby reinforcing the structure more efficiently. This was followed by the analysis of the UV treated hair samples. It can be seen that the tensile strength of control decreases slightly after exposure to UV light. However, the MD and MD/TA coated hair strands demonstrate almost similar result as before exposure to UV light showing its capacity for UV blocking and mechanical enhancement. Following mechanical strength analysis, the antistatic properties of the MD and MD/TA treated hair fibers were investigated to evaluate surface charge dissipation capability of the nanocomplex. To assess the antistatic properties, nanocomplex-treated and untreated hair samples were rubbed with a balloon, and their antistatic behavior was observed before and after exposing to UV irradiation (Fig. 3b). The before and after UV irradiated control hair strands adhered readily to the balloon surface due to static charge accumulation. In before and after UV irradiated hair treated with MD, a partial reduction in electrostatic accumulation was observed, which was attributed to the surface charge originating from Lys-derived amine groups within the MD. However, in the case of before and after UV irradiated MD/TA-treated hair, the MD/TA complex effectively suppressed the accumulation of charge on the hair surface. This was due to an increase in the surface conductivity, based on the electron mobility and polyphenol structure of tannic acid. Therefore, MD/TA demonstrated superior antistatic performance, indicating an improvement in the charge dissipation properties. In addition to the mechanical strength analysis and antistatic performance, morphological changes in the hair strands were also analyzed to directly visualize the protective effect of MD/TA under UV irradiation. The microstructural changes in the hair samples before and after UV irradiation were examined using SEM (Fig. 3c). Before UV exposure, all the samples exhibited relatively intact cuticle layers. However, after UV irradiation, the control sample showed pronounced surface roughening and cuticle damage. Although minor cuticle damage was still observed in the MD-treated samples, the hair treated with MD/TA, in contrast, exhibited a smoother surface and maintained a more intact cuticle structure. This shows the effect of efficient coating on the MD/TA coated samples owing to various hydrogen bonding and hydrophobic interactions with the hair proteins and its UV protection capacity *via* damage repair utilizing Michael addition reaction between the galloyl groups of MD/TA and -SH bonds of the damaged cuticles. The UV blocking capacity of the MD/TA nanocomplex was further analyzed using the Ellman assay. The results, as shown in Fig. S13, demonstrated the presence of a high-amount of -SH bonds caused by the breakage of -S-S- bonds in keratin of hair strands under the application of UV light. This was in contrast to the MD and MD/TA treated hair samples as seen from the very low TNB concentration value. This may have been caused by the presence of galloyl groups in TA which convert to their quinone structure (in presence of ROS generated by UV irradiation) and undergoes Michael addition reaction with the -SH bonds in the UV exposed hair strands attributing to the formation of a uniform and tightly adhered coating layer that serves as a physical barrier, thereby mitigating structural degradation caused by UV radiation. These observations were further verified using XPS analysis of the hair strands for control and MD/TA coated samples. The results in Fig. S14, clearly shows the decrease of -S-S-, -S-H and -S-C peak area % in MD/TA samples signifying the aforementioned interactions taking place in the repair process. Finally, a comprehensive study was carried out for comparing various other nanoplatforms with UV blocking capability with the MD/TA nanocomplex. From Table S1 it can be seen that the nanocomplex demonstrated UV blocking in the UVA and UVB region while other platforms showed UV blocking only in the UVA region [[47], [48], [49]]. Additionally, the nanocomplex was fabricated with an application dedicated towards hair protection, damage repair and hair strengthening as compared to most of the other platforms in literature [[50], [51], [52]].

### 3.3 Antioxidant property of MD/TA nanocomplex

**Fig. 4:**
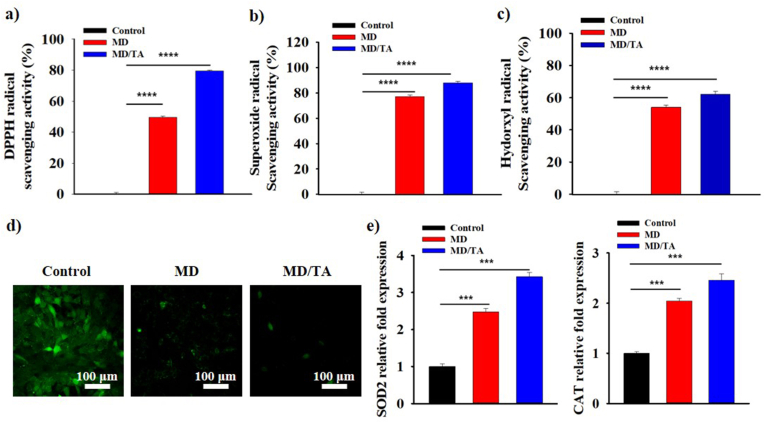
**ROS Scavenging activity and antioxidant gene expression.** (**a**) DPPH radical scavenging, (**b**) superoxide radical, and (**c**) hydroxyl radical scavenging activity of MD and MD/TA. (**d**) ROS-scavenging assessment with H_2_-DCFH-DA assay, and (**e**) expression levels of antioxidative signaling genes: *SOD2*, and *CAT* genes of 3T3-L1 cell lines after treatment with control (only cell), MD and MD/TA. All analysis was carried out for n = 3 samples. An unpaired Student's t-test was used for statistical analysis. ∗p < 0.05; ∗∗p < 0.01; ∗∗∗p < 0.001; ∗∗∗∗p < 0.0001; ns indicates non-significant differences.

The excessive production of ROS in hair caused by UV exposure significantly contributes to oxidative damage, highlighting the critical role of antioxidant systems in effective hair protection. In this study, melanoidin (MD) and tannic acid (TA), both possessing inherent ROS scavenging properties, were combined to synthesize the MD/TA nanocomplexes. The antioxidant efficacy was assessed using DPPH, hydroxyl radical (•OH), and superoxide radical (O_2_^•–^) scavenging assays (Fig. 4a–c). Across all three assays, MD/TA consistently exhibited greater scavenging activity than MD, indicating an enhancement in antioxidant capacity. This improvement is attributed to the incorporation of TA, a polyphenolic compound containing multiple hydroxyl groups that can donate electrons or hydrogen atoms to neutralize free radicals, thereby enhancing the antioxidant properties of the composite. Following simulated radical scavenging analyses, the *in vitro* antioxidative capability of MD and MD/TA was conducted in the presence of 3T3-L1 cells. The cells were first treated with H_2_O_2_ to induce ROS generation and subsequently treated with MD or MD/TA to evaluate their ability to mitigate intracellular ROS with the help of DCFH-DA staining assay (Fig. 4d). Both MD and MD/TA exhibited antioxidant effects, with MD/TA showing a more pronounced ROS-scavenging capacity. In addition to ROS mitigation at the cellular level, the antioxidative activity of MD and MD/TA was investigated to assess its capability to affect necessary antioxidant genes through qRT-PCR analysis. The expression levels of antioxidant-related genes, *SOD2* and *CAT*, were analyzed (Fig. 4e). MD/TA treatment resulted in a greater induction of gene expression compared to MD, thus confirming the enhanced antioxidant efficacy of MD/TA owing to the presence of TA.

### 3.4 Biocompatibility of MD/TA nanocomplex

**Fig. 5:**
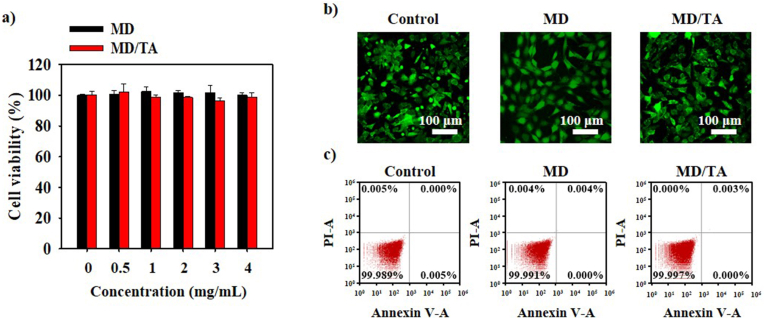
***In vitro* biocompatibility evaluation of MD and MD/TA nanocomplexes.** (**a**) MTT assay, (**b**) live and dead assay, (**c**) FACS analysis depicting 3T3-L1 cell apoptosis and necrosis after treatment with control (only cell), MD, and MD/TA.

The biocompatibility of MD and MD/TA was assessed at various concentrations using 3T3-L1 precursor cells. After a 24 h treatment with MD or MD/TA at concentrations ranging from 0 to 4 mg/mL, high survival rates were observed across all concentrations, indicating minimal toxicity (Fig. 5a). These findings indicate that both substances exhibit excellent biocompatibility. Furthermore, in a Live/Dead assay utilizing CLSM, the majority of cells demonstrated positive survival staining (Calcein-AM), with minimal indications of cell death (Fig. 5b). Furthermore, FACS-based apoptosis and necrosis analysis confirmed that neither early nor late apoptosis or necrosis was induced, thereby corroborating the stability of the MD and MD/TA complex at the cellular level (Fig. 5c). Taken together, these findings establish that MD and MD/TA nanocomplexes demonstrate excellent biocompatibility and high cellular viability, across multiple *in vitro* analysis demonstrating its biosafety and scope for applicability in future cosmetic applications.

### 3.5 In vitro analysis of UV blocking activity

**Fig. 6:**
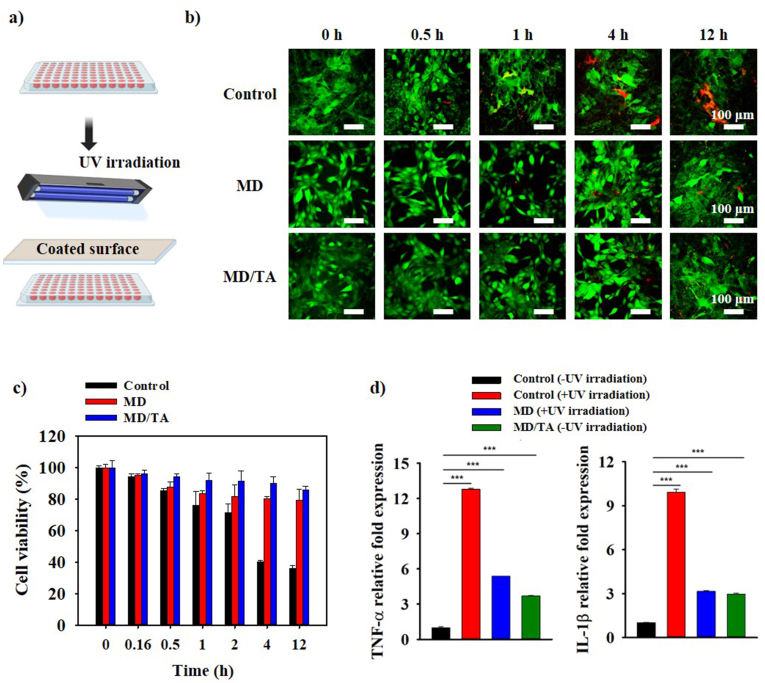
**Protective Effects of MD and MD/TA Coated Surfaces Against UV-Induced Cellular Damage in 3T3-L1 Cells.** (**a**) Schematic of the *In vitro* experimental setup for UV blocking activity: 3T3-L1 cells were seeded and exposed to UV irradiation using MD- or MD/TA-coated PET films. (**b**) Live/dead staining of cells, and (**c**) cell viability assessed by MTT assay at various UV irradiation times with control (PET film), MD, and MD/TA coated surface. (**d**) qRT-PCR analysis of *TNF-α* and *IL-1β* expression levels after treatment with control (PET film), MD, and MD/TA coated surface. All analysis was carried out for n = 3 samples. An unpaired Student's t-test was used for statistical analysis. ∗p < 0.05; ∗∗p < 0.01; ∗∗∗p < 0.001; ∗∗∗∗p < 0.0001; ns indicates non-significant differences.

Following the culture of 3T3-L1 cells, the UV-blocking efficacy was assessed by overlaying MD- or MD/TA-coated PET films above the cells and subjecting them to UV radiation in dark conditions (Fig. 6a) [53,54]. Live/Dead confocal analysis (Fig. 6b) demonstrated a significant increase in cell death in the control group following prolonged UV exposure, whereas cell viability was notably preserved on both MD- and MD/TA-coated surfaces. Notably, the MD/TA-coated group exhibited superior protective performance as compared to MD, which can be ascribed to the strong UV-absorbing conjugated aromatic structure of tannic acid. To further substantiate these findings, quantitative cell viability was analyzed using MTT assay. The results (Fig. 6c) demonstrated that the MD/TA group provided better protection to the cells as shown by the significantly higher cell viability in comparison to both the MD-treated and control groups. Subsequently, FACS analysis demonstrated a marked reduction in UV-induced apoptosis in the MD/TA group, surpassing that of MD treatment, while the control group exhibited pronounced apoptotic activity (Fig. S15). Additionally, the qRT-PCR analysis of inflammatory gene markers *TNF-α* and *IL-1β* revealed that UV-induced inflammatory responses were most effectively suppressed in the MD/TA-coated group (Fig. 6d) in comparison to control and MD coated groups. These observations demonstrate the photoprotective effect of MD/TA against UV-mediated cellular damage on various inflammatory genes. Moreover, these findings support the potential of MD/TA as a functional photoprotective material for protecting keratin-rich tissues such as hair and skin from ultraviolet radiation, suggesting its future application as a bio-based coating or cosmetic ingredient for UV protection and anti-inflammatory purposes.

Following the *in vitro* analysis of the MD/TA with 3T3-L1 cells, the nanocomplex and its potential for use in future cosmetic and hair-based application was experimented. For this purpose, human dermal papilla cells (DPCs) were used in the analysis. The quantitative biocompatibility results as shown in Fig. S16a showed the high biocompatibility of the MD/TA at various concentration using MTT. Similarly, the quantitative UV blocking analysis showed close to 90 % viability of the DPCs treated with MD and MD/TA (Fig. S16b) as compared to control (only cells) which hinted at its potential for UV protection. This was followed by observing the live and dead assay using CLSM optical images. As shown in Fig. S17, the DPCs remained alive in presence of MD and MD/TA cells which prompted us to check its ROS scavenging capability. The H_2_O_2_ induced DPCs were treated with MD and MD/TA and stained with H_2_-DCFH-DA dye. As seen in Fig. S18, the presence of H_2_O_2_ induced ROS in the DPCs which stained the cells bright green. However, in the presence of MD, the brightness decreased and, in the presence, of MD/TA all the remaining ROS was quenched and there was no green FL observed. This was caused by the presence of numerous galloyl and catechol groups in the MD/TA which helped to neutralize the induced ROS in DPCs. Finally, the UV blocking potential was observed as the DPCs were irradiated with UV light (∼365 nm) for various time (0, 0.5, 1, 4 and 12 h) which showed the decrease in the cell viability from 4 h to 12 h as seen in Fig. S19. However, in the presence of UV protecting MD and MD/TA the DPCs are well protected and shielded from the harmful UV rays which is shown by the high viability of the DPCs. This shows that the MD/TA nanocomplex is capable of being used in future hair-based cosmetic application with potential for protecting hair from UV rays, and capacity for neutralizing damage from ROS.

## 4 Conclusion

In this study, a melanoidin/tannic acid (MD/TA) nanocomplex was successfully synthesized and evaluated as a dual-functional material for hair protection and mechanical reinforcement with UV-protective and antioxidant properties. Compared with melanoidin, the MD/TA nanocomplex exhibited superior performance in protection against UV radiation and the scavenging of reactive oxygen species (ROS). This enhancement arises from the synergistic interaction between the polyphenolic structure of tannic acid and the light-absorbing properties of melanoidin, which together provide broad-spectrum UVA/UVB protection while concurrently suppressing intracellular ROS generation. The structural stability and biocompatibility of MD/TA were confirmed through comprehensive physicochemical and biological assessments, including optical analysis, surface charge evaluation, electrochemical characterization, antioxidant assays, and cytotoxicity tests. The excellent photoprotective efficacy of the MD/TA nanocomplex was validated in presence of 3T3-L1 cells by demonstrating improved cell viability and suppression of inflammatory gene expression following exposure to UV irradiation. Moreover, in the presence of human derived papilla cells (DPCs) the MD/TA nanocomplex showed its efficient photo-protectiveness, biocompatibility and ROS scavenging efficacy which helped modulated inflammatory cytokines. These observations collectively ensure the applicability of the MD/TA nanocomplex in both research and commercial hair-care-based future applications. Finally, these findings help to establish MD/TA as a promising bio-derived photoprotective platform capable of safely mitigating UV-induced tissue damage and oxidative stress.

## Data availability statement

The data will be made available upon request.

## CRediT authorship contribution statement

**Woo Il Lee:** Conceptualization, Data curation, Formal analysis, Investigation, Methodology, Visualization, Writing – original draft. **Tae Min Kim:** Data curation, Formal analysis, Investigation, Methodology, Writing – original draft. **Sanghee Lee:** Data curation, Formal analysis, Methodology, Writing – review & editing. **Gibaek Lee:** Data curation, Methodology, Resources, Supervision, Validation, Writing – review & editing. **Sung Young Park:** Conceptualization, Data curation, Formal analysis, Funding acquisition, Methodology, Project administration, Resources, Supervision, Validation, Writing – original draft.

## Declaration of competing interest

The authors declare that they have no known competing financial interests or personal relationships that could have appeared to influence the work reported in this paper.

## Data Availability

Data will be made available on request.
