# Supplementary material for: Bioinspired Melanoidin–Polyphenol nanocomplex for hair protection and mechanical reinforcement with antioxidant activity
Source: Mater Today Bio. 2026 May 25;38:103277. doi: 10.1016/j.mtbio.2026.103277 (PMC13240830; doi:10.1016/j.mtbio.2026.103277)
Supplement: Multimedia component 1 [file mmc1.docx]

**Supporting Information**

**Bioinspired Melanoidin–Polyphenol Nanocomplex for Hair Protection and Mechanical Reinforcement with Antioxidant Activity**

*Woo Il Lee ^a,1^, Tae Min Kim ^a,1^, Sanghee Lee*^c^*, Gibaek Lee^b*^, Sung Young Park^a,c *^*

^a^Department of IT and Energy Convergence, Korea National University of Transportation, Chungju 27469, Republic of Korea

^b^Department of Chemical and Biological Engineering, Korea National University of Transportation, Chungju 27469, Republic of Korea

^c^School of Nanomedical Engineering, Korea National University of Transportation, Chungju 27469, Republic of Korea

***Corresponding author:**

Tel: +82-(0)43-841-5225, Fax: +82-(0)43-841-5220, E-mail: glee@ut.ac.kr (G. Lee), [parkchem@ut.ac.kr](mailto:parkchem@ut.ac.kr) (Sung Young Park).

^1^ These authors contributed equally.

**
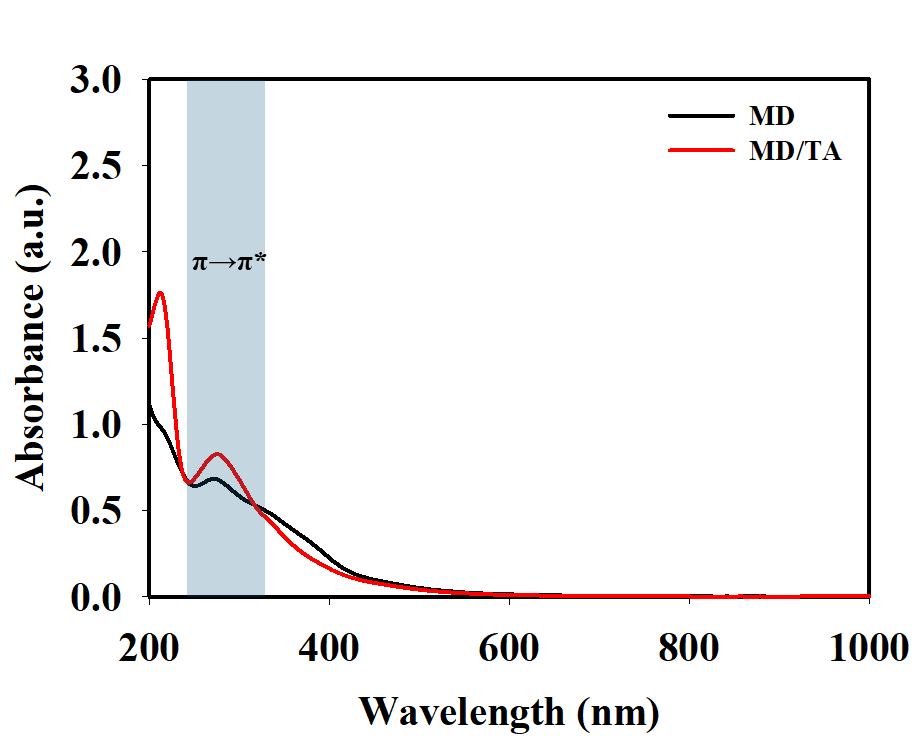
**

**Figure S1.** The UV-vis spectra of the MD and MD/TA UV blocking nanocomplex at a concentration of 0.05 mg/mL. All samples analyzed for n =3 samples.

**
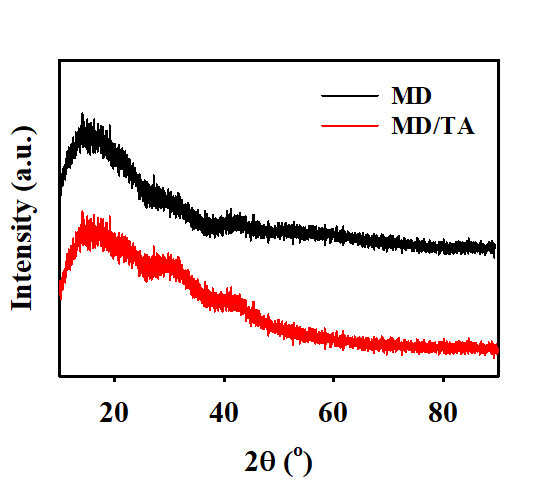
**

**Figure S2.** X-ray diffraction (XRD) spectra of MD and MD/TA.

**Figure S3.** Thermal gravimetric analysis (TGA) measurement of MD and MD/TA

**
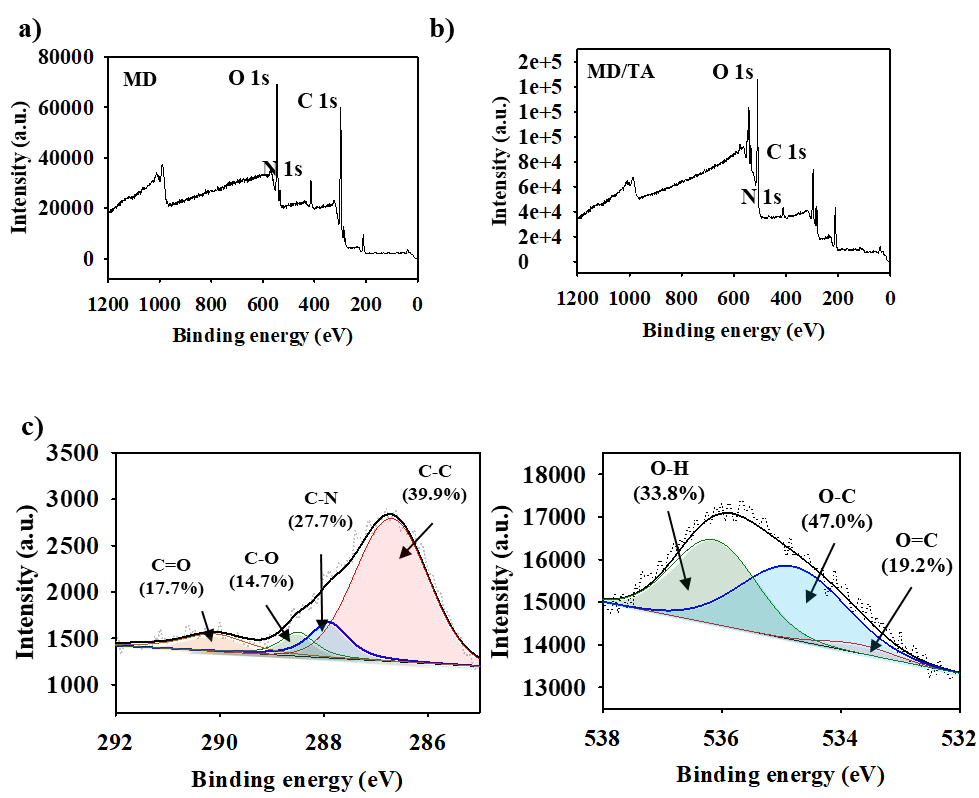
**

**1**

**2**

**3**

**4**

**Figure S4.** XPS survey scan of, (**a**) MD, and (**b**) MD/TA, and (**c**) narrow scan (C1s and O1s) of MD.


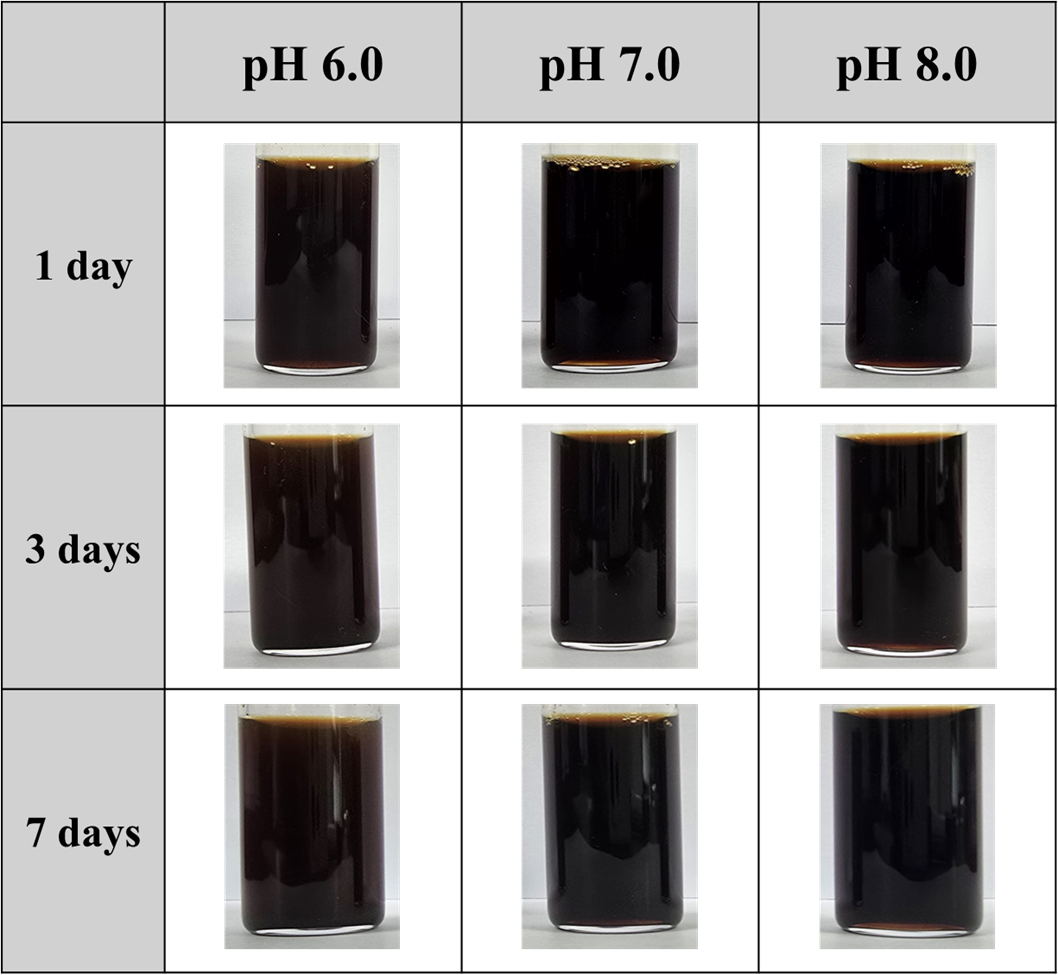


**Figure S5.** Colloidal stability analysis of the MD/TA in presence of PBS pH 6.0, 7.0 and 8.0 to ascertain the successful formation of the nanocomplex. All samples analyzed for n =3 samples. Sample concentration is 10 mg/mL.


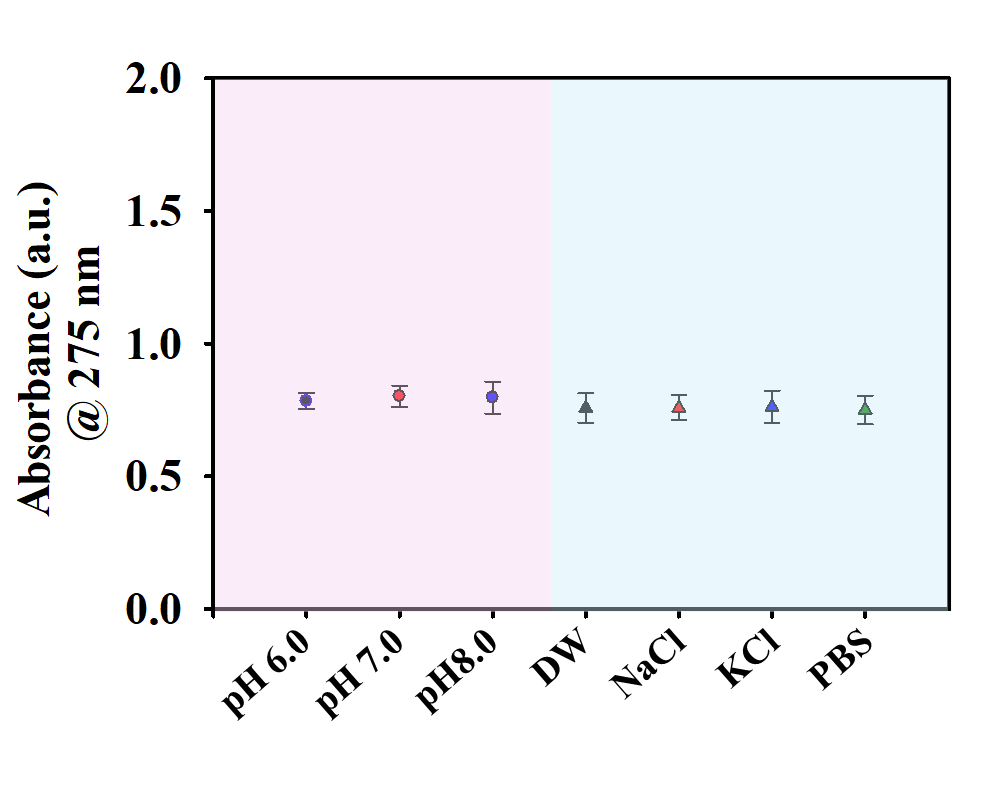


**Figure S6.** The colloidal stability of the MD/TA nanocomplex by visualizing any shifts in the absorbance intensity of the UV spectrum at 275 nm wavelength (MD/TA UV peak) after exposure to various pH and ionic compounds. Concentration of samples for checking UV was 0.05 mg/mL All samples analyzed for n =3 samples.

**
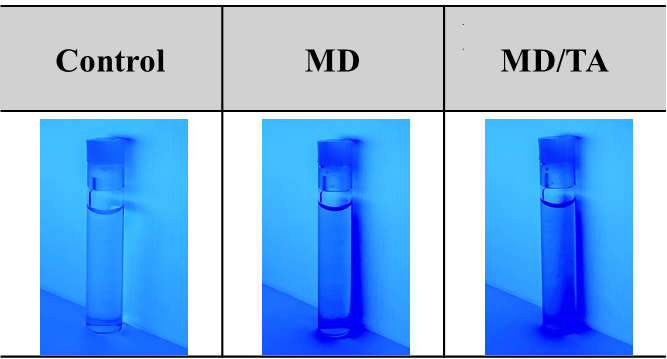
**

**Figure S7.**  Photographs of the UV protection properties of MD and MD/TA in the presence of UV light. All samples analyzed for n =3 samples.


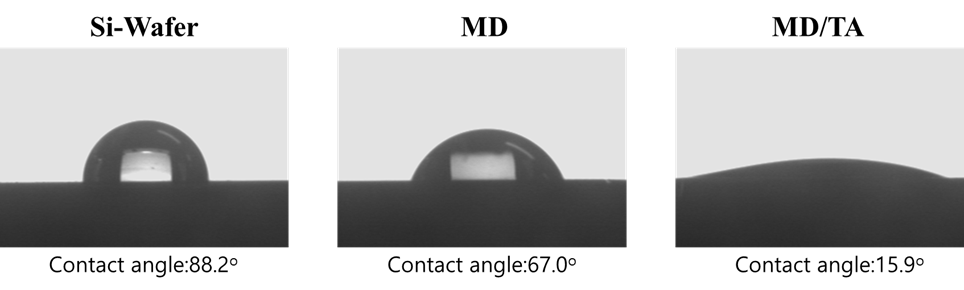


**Figure S8.**  Optical images of the contact angles of bare Si-wafer, MD and MD/TA after coating on Si-wafer. All samples analyzed for n =3 samples.


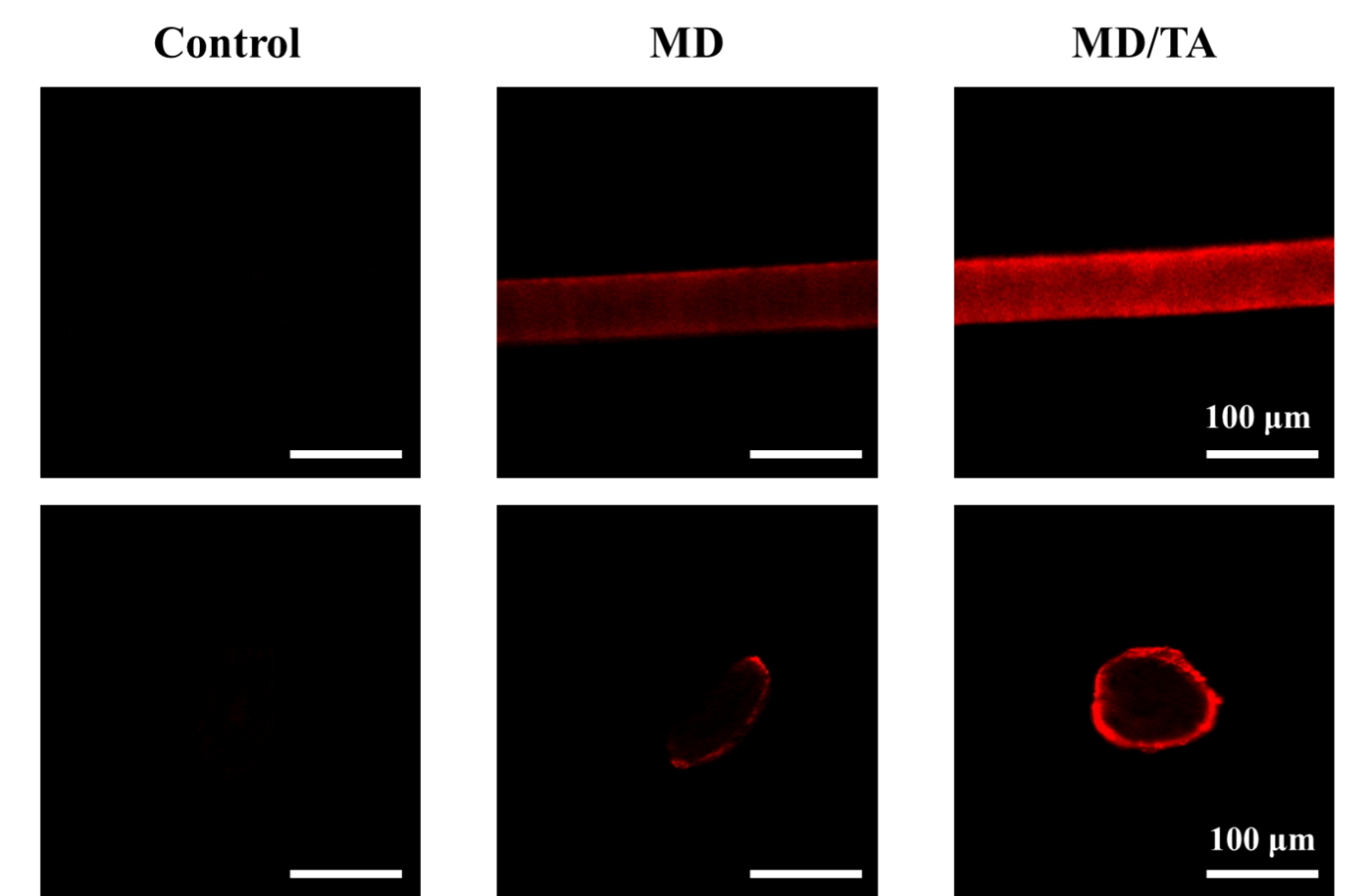


**Figure S9.** The CLSM optical images of the control and MD and MD/TA coated hair strands with the MD and MD/TA being mixed with Rhodamine B dye to track the efficiency of their surface coating ability. All samples analyzed for n =3 samples.


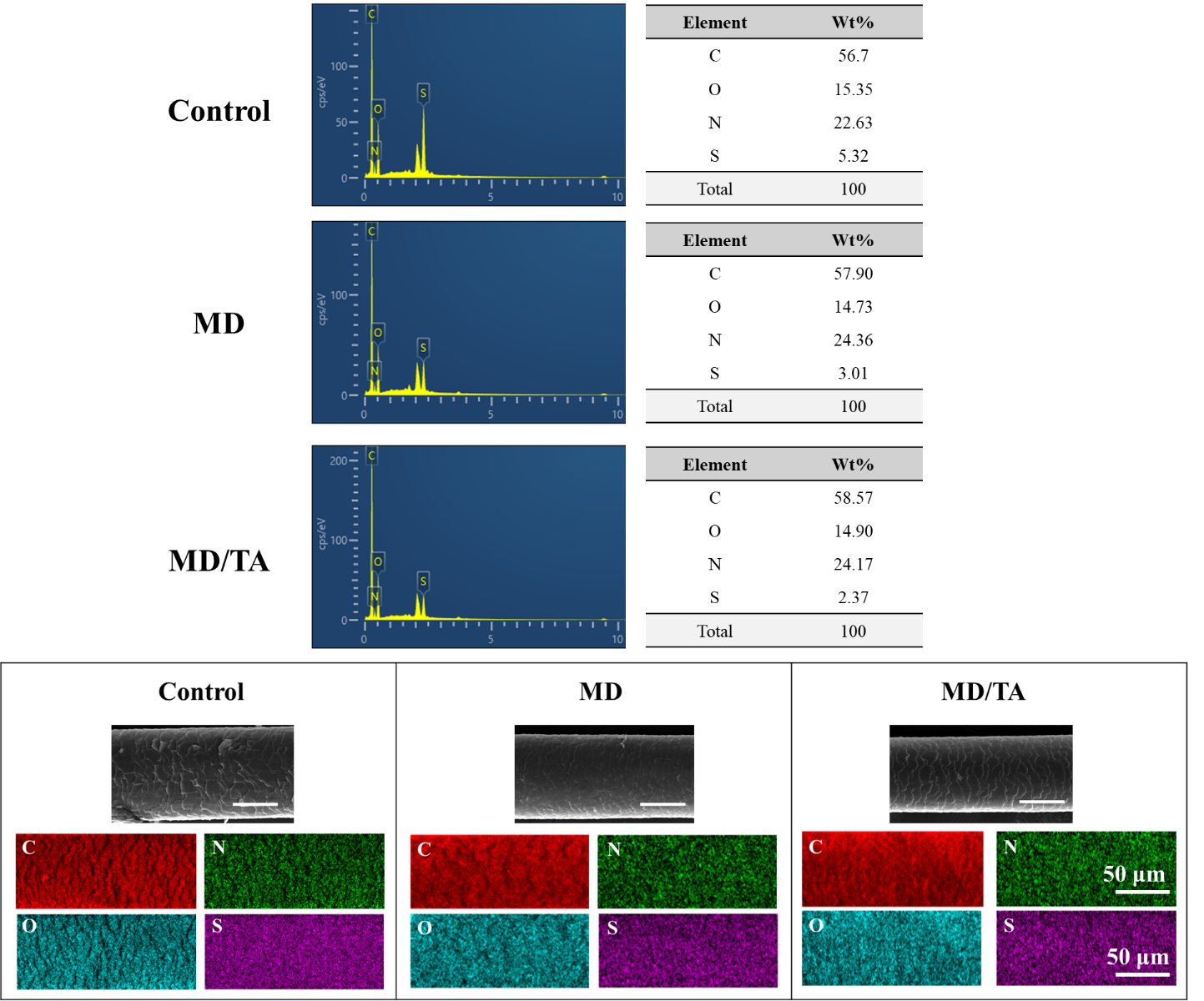


**Figure S10.** Elemental mapping, EDX spectra of the control sample, MD and MD/TA coated hair samples before and after UV treatment.


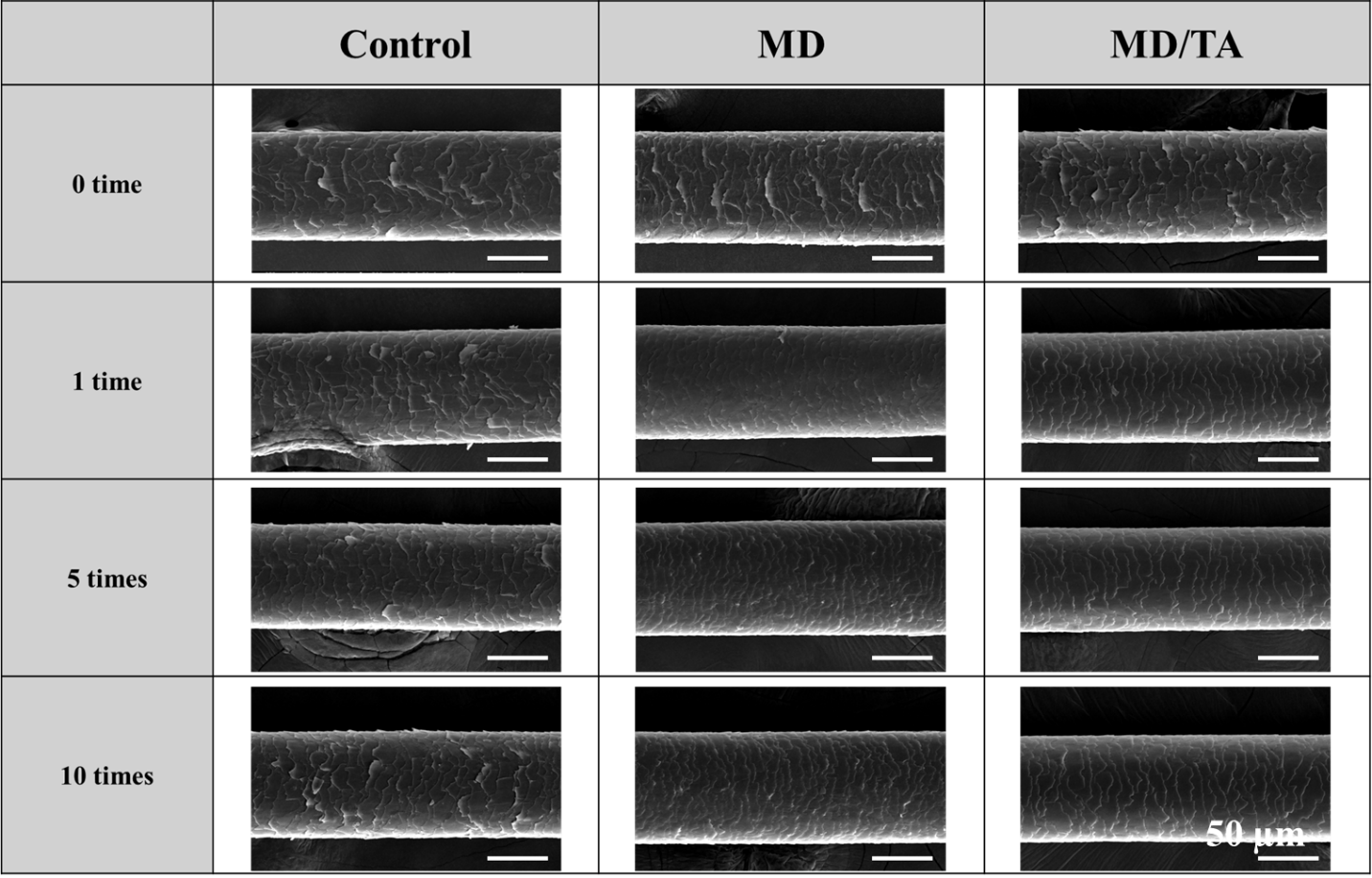


**Figure S11.** The coating durability of MD and MD/TA after rinse test for 10 cycles analyzed using SEM images (*Magnification: 50 µm*). All samples analyzed for n =3 samples.


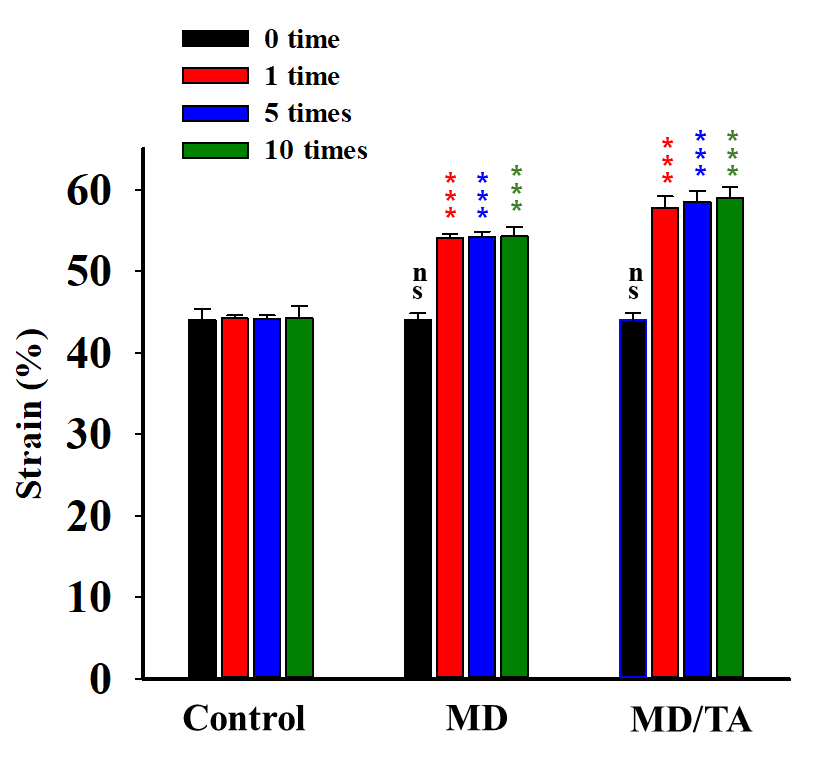


**Figure S12.** The coating durability of MD and MD/TA after rinse test for 10 cycles which was analyzed using UTM mechanical analysis. All samples analyzed for n =3 samples. An unpaired Student's t-test was used for statistical analysis. *p < 0.05; **p < 0.01; ***p < 0.001; ****p < 0.0001; ns indicates non-significant differences.


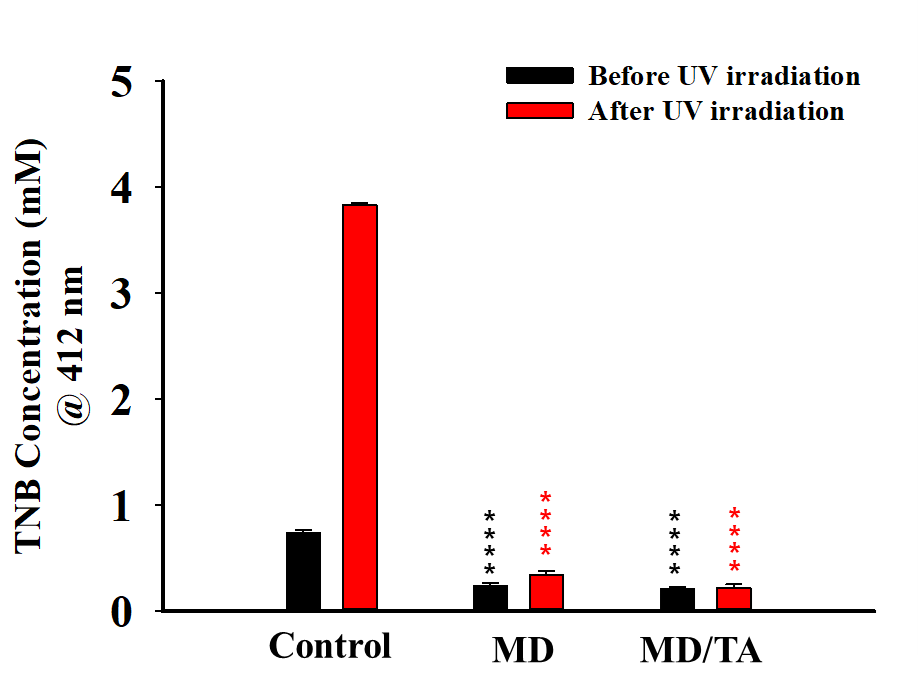


**Figure S13.** The Ellman assay of UV (~365 nm, 1 day) irradiated hair samples for control (untreated), MD and MD/TA results with corresponding images of the sample color change. Analysis was carried out for n = 3 samples. An unpaired Student's t-test was used for statistical analysis. *p < 0.05; **p < 0.01; ***p < 0.001; ****p < 0.0001; ns indicates non-significant differences.


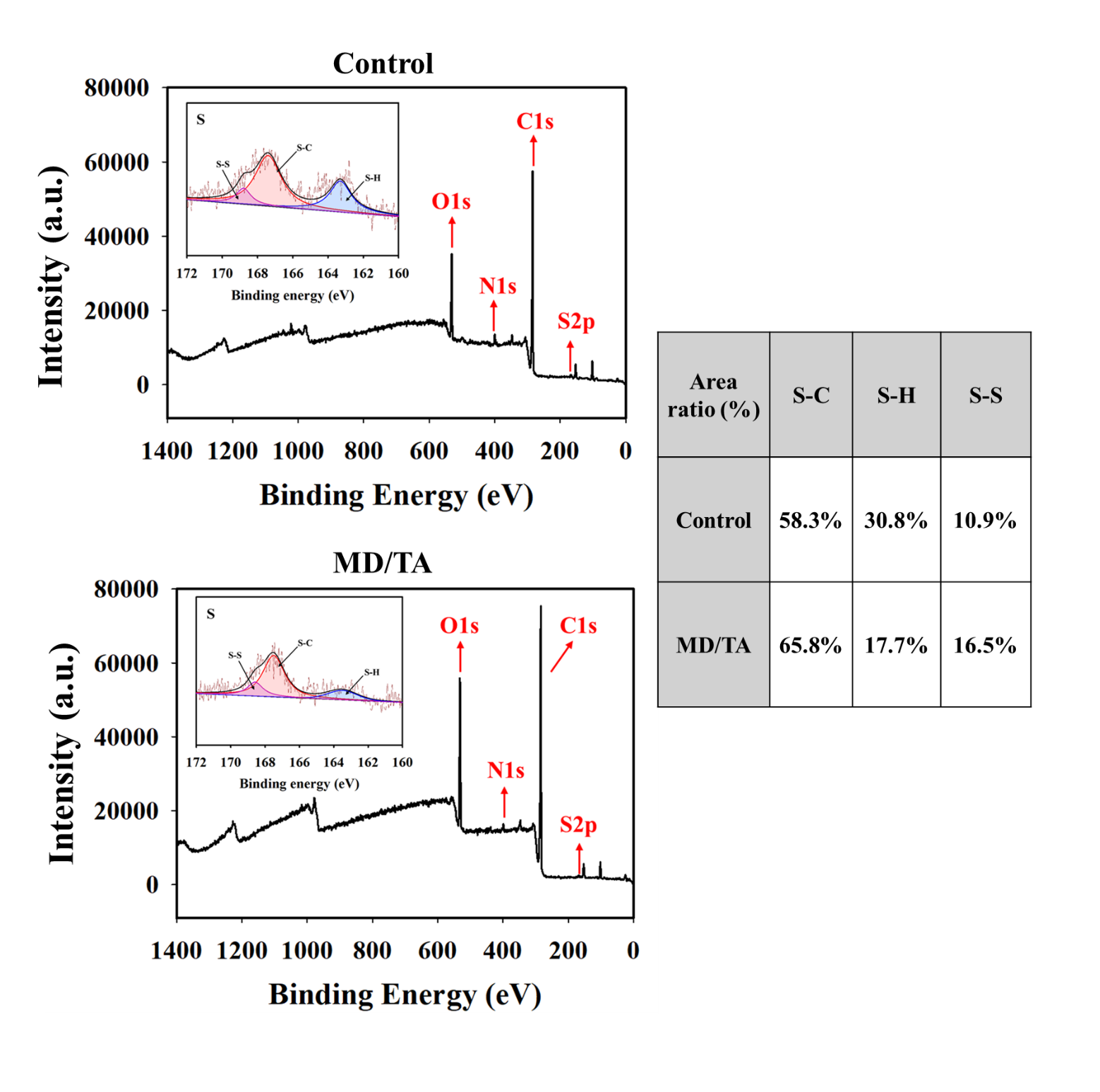


**Figure S14.** XPS survey and narrow scan of control and MD/TA coated hair strands showing the shift of intensity of -S-H and -S-C bonds demonstrating UV blocking and repair mechanism. Additionally, the area ratio %, quantitatively verifies the change in area for the -S-S-, -S-H, and -S-C- bonds.


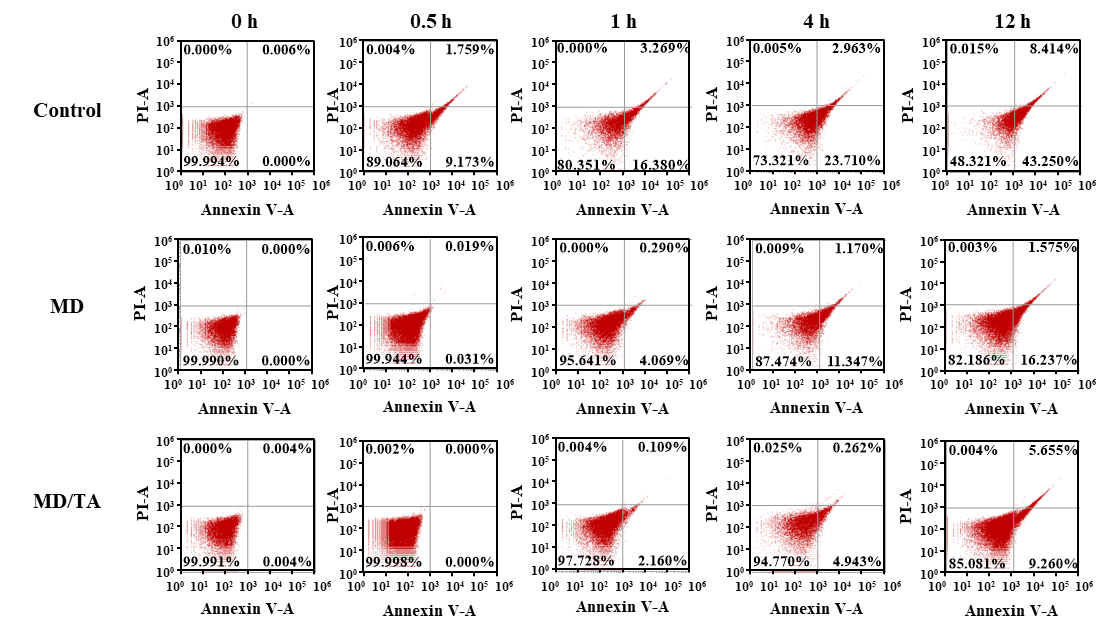


**Figure S15.** FACS analysis depicting 3T3-L1 cell apoptosis and necrosis with MD and MD/TA coated surfaces, depending on UV irradiation time. All samples analyzed for n =3 samples.


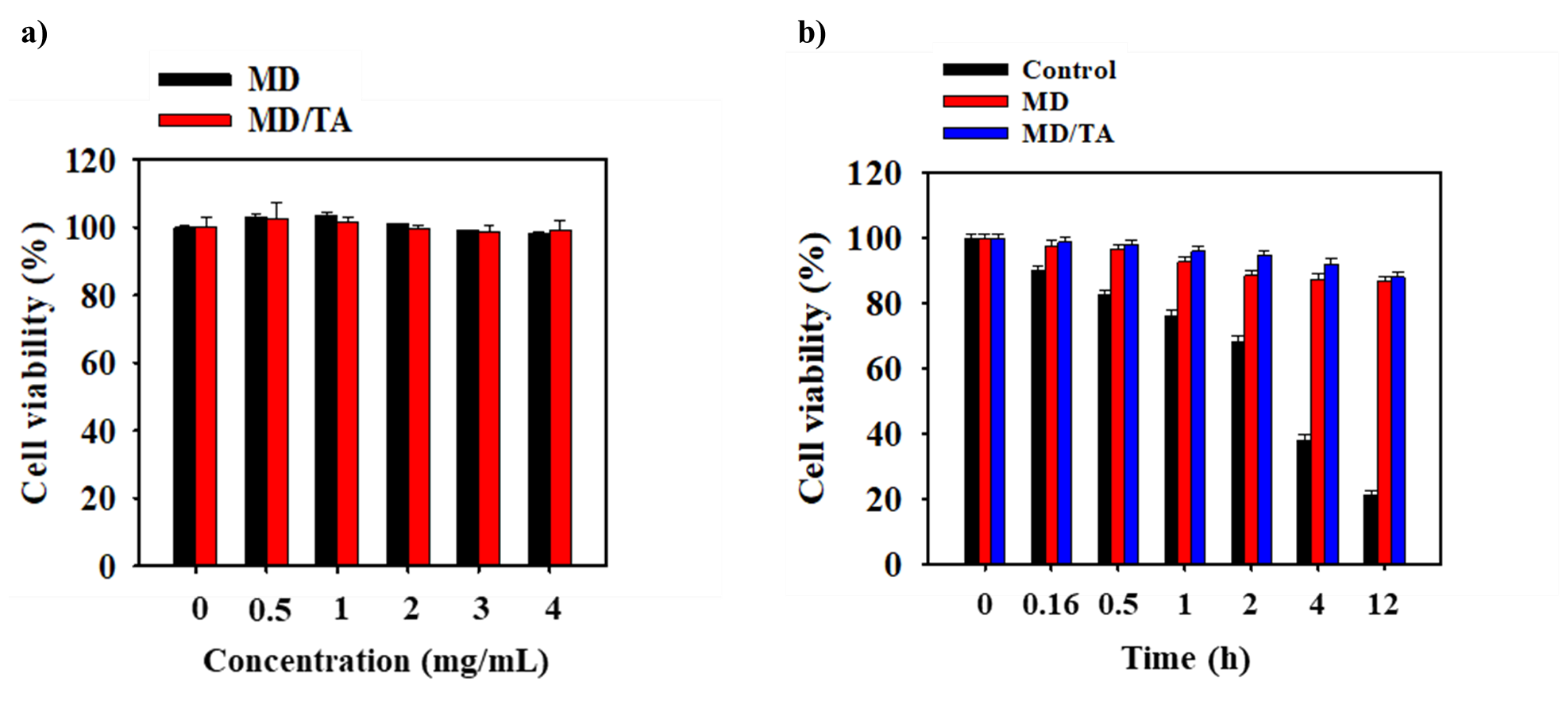


**Figure S16.** (**a**) The quantitative cytotoxic effect of MD and MD/TA on the DPCs (dermal papilla cells) (concentration: 0.5 mg/ mL), and (**b**) the quantitative biocompatibility for analyzing the *in vitro* UV blocking effect of MD and MD/TA (concentration: 1 mg/ mL) on the DPCs after UV (~365 nm) irradiation for 0, 0.16, 0.5, 1, 2, 4 and 12 h using microplate reader. All samples were analyzed for n = 3 samples.


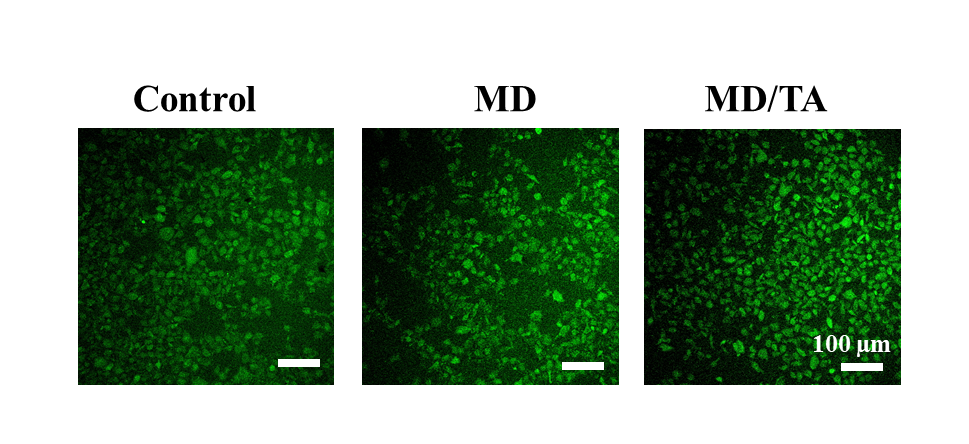


**Figure S17.** The *in vitro* biocompatibility of the MD and MD/TA samples in presence of DPCs was analyzed using live and dead assay (*Magnification: 100 µm*). All samples were analyzed for n = 3 samples.


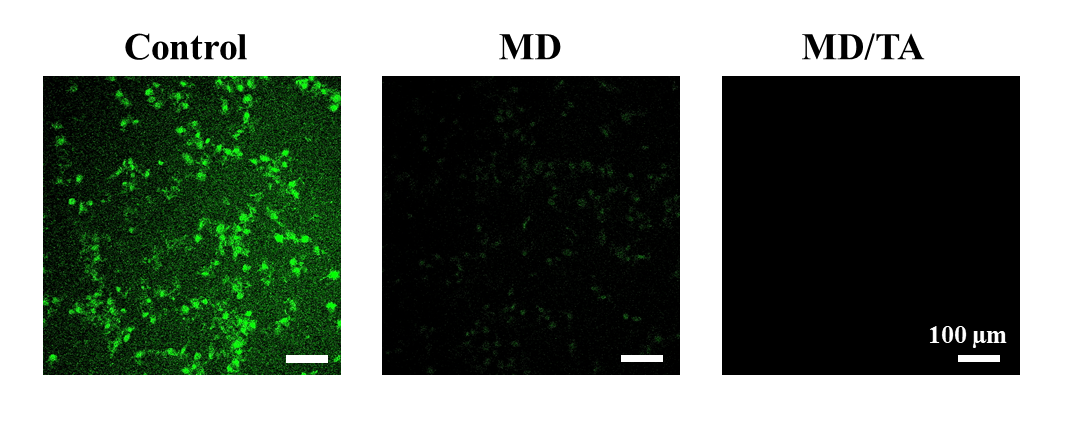


**Figure S18.** The ROS scavenging efficiency of MD and MD/TA in presence of DPCs after inducing ROS via treatment with 300 µM H_2_O_2_ for 2 h. The green color generated is owing to the ROS staining dye H_2_-DCFH-DAwhich stains intracellular ROS with green Fl (*Magnification: 100 µm*). All samples were analyzed for n = 3 samples.


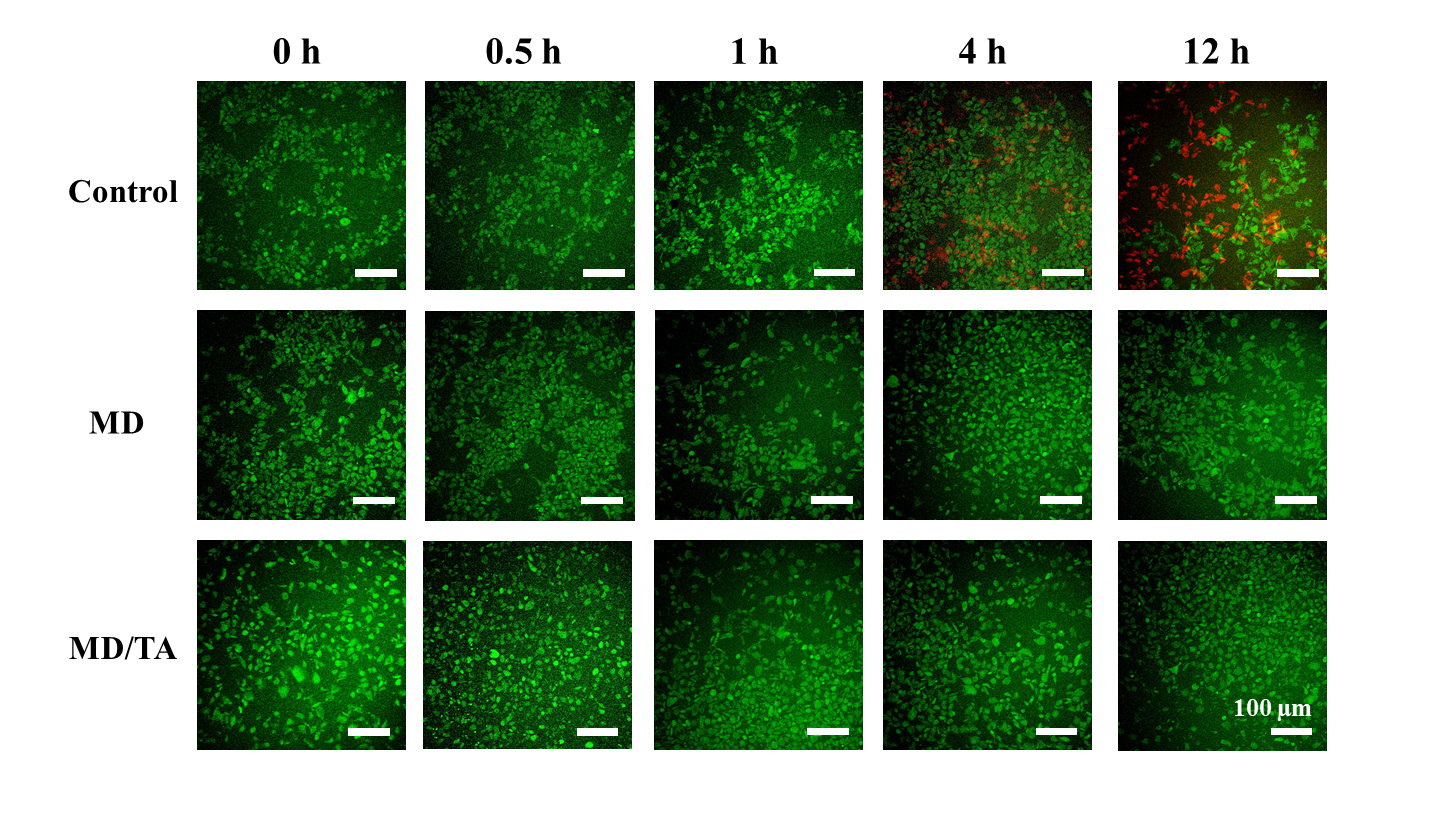


**Figure S19.** Additionally, the UV blocking effect of the MD and MD/TA on the DPCs were analyzed by treating DPCs with UV (~365 nm) irradiation for 0, 0.5, 1, 4 and 12 h using and observing under CLSM using live and dead assay (*Magnification: 100 µm*). All samples were analyzed for n = 3 samples.

**Table S1.** Comparison of MD/TA nanocomplex and its UV blocking performance as compared to other similar published methods in literature.

| Platform | Damage repair | Interaction for repair | UV absorption region | In vitro model | Application | Reference |
| --- | --- | --- | --- | --- | --- | --- |
| Squid ink melanin nanoparticles | Yes | Ionic coordination | 290 – 400 nm | HaCaT cells | Bio-based hair colorant | [47] |
| TA/TiO_2_ nanoparticles | No | - | 290 - 400 nm | - | Anti-UV skin protection | [48] |
| (PC-PDA@TiO_2_) nanoparticles | No | - | 290 - 320 nm | 3T6 cells | Sunscreen product | [49] |
| Nanostructured lipid carriers (NLCs) | Yes | Hydrophobic interaction | 280 - 400 nm | - | Anti-hair damage and protection cream | [50] |
| Natural melanin-TiO_2_ (NM/TiO_2_) nanohybrids | No | - | 280 - 400 nm | - | UV shielding Films | [51] |
| Chromium-doped hydroxyapatite nanoparticles | No | - | 280 – 320 nm | - | UV blocking cream | [52] |
| MD/TA nanocomplex | **Yes** | **ROS scavenging/ Michael addition** | **280-400 nm** | **3T3-L1, murine preadipocyte/**  **DPCs (Dermal papilla cells)** | **Hair care/ UV protection** | **This work.** |
